# Multimodal Assessment of Consciousness with Brain-Computer Interfaces and Artificial Intelligence: From Acquired Brain Injury to Neurodegenerative Disease

**DOI:** 10.3390/jcm15145398

**Published:** 2026-07-09

**Authors:** Bernard Kordas

**Affiliations:** Department of Human Physiology and Pathophysiology, School of Medicine, Collegium Medicum, University of Warmia and Mazury, 10-082 Olsztyn, Poland; bernard.kordas@uwm.edu.pl

**Keywords:** disorders of consciousness, neurodegenerative disease, acquired brain injury, covert cognition, multimodal assessment, brain–computer interface, artificial intelligence, functional neuroimaging, autonomic measures, cognitive-motor dissociation

## Abstract

The assessment of consciousness has been shaped largely by research on acquired disorders of consciousness after acute or chronic brain injury, but similar problems of unreliable behavioral expression increasingly arise in neurodegenerative disease. This translational overlap is especially relevant when preserved cognition, awareness, or intentionality cannot be reliably expressed because of severe motor impairment, fluctuating arousal, cognitive decline, aphasia, apraxia, or impaired cooperation. In neurodegenerative disease, degeneration of arousal systems, large-scale brain networks, cognition, and motor pathways may similarly make observable behavior an unreliable measure of awareness. The challenge is not only to determine if a patient responds, but also to ask if residual awareness, intentionality, or covert cognition can still be detected through physiological signals. This review discusses how contemporary modalities reshape this assessment. Electroencephalography has moved from a descriptive measure of background activity to a bedside tool capable of probing event-related responses, network organization, and cortical complexity. Magnetic resonance methods reveal altered connectivity within thalamocortical and default mode network systems, while functional near-infrared spectroscopy adds a portable hemodynamic approach that may be repeated at the bedside and integrated with active paradigms. Brain–computer interfaces provide a translational step by converting neural responses into evidence of command following or, in selected patients, into communication, and artificial intelligence strengthens these approaches by extracting clinically meaningful patterns from complex neural and hemodynamic data. Additionally, autonomic measures, including heart rate variability and baroreflex indices, are considered as auxiliary physiological context for arousal and engagement, and not as direct markers of awareness. Because the most mature evidence for covert awareness and cognitive-motor dissociation comes from acquired disorders of consciousness, this review treats brain injury literature as a methodological foundation instead of as directly interchangeable evidence for neurodegenerative disease. It then examines how these approaches may be adapted to neurodegenerative contexts, especially ALS, severe dementia, Lewy body disease with fluctuating cognition, and conditions in which communication or motor output becomes unreliable.

## 1. Introduction

Disorders of consciousness are classically conceptualized through impaired wakefulness, impaired awareness, or dissociation between the two, and they remain among the most difficult states to characterize when motor output is limited or unreliable [1,2,3]. In neurodegenerative disease, the problem often appears in a less stereotyped but equally important form, because degeneration can disrupt attention and awareness of the self, whereas motor neurodegenerative disease can limit the capacity to express preserved cognition through behavior [4,5,6]. Fluctuations in attention and awareness are prominent in dementia with Lewy bodies and can distinguish it clinically from Alzheimer’s disease and vascular dementia [7,8]. Neuroimaging studies in Lewy body dementia have linked changes in regional perfusion with hallucinations and fluctuations in consciousness, which supports a biological understanding of these phenomena, not a purely descriptive one [9]. In severe Alzheimer’s disease, awareness may persist in forms that are difficult to capture by routine bedside assessment, and multimodal approaches combining electrophysiology and imaging may provide physiological and imaging measures that may support assessment [10].

The present review deliberately bridges two related but clinically distinct areas. Acquired disorders of consciousness after acute or chronic brain injury provide the strongest methodological foundation for detecting covert awareness, cognitive-motor dissociation, and command-following when behavior is absent or inconsistent. Neurodegenerative diseases raise a partly different but increasingly important translational problem: cognition, arousal, attention, language, motor output, and autonomic regulation may deteriorate gradually and unevenly, making behavioral assessment progressively less reliable. Therefore, evidence from acquired brain injury is not treated here as directly transferable to Alzheimer’s disease, Parkinsonian syndromes, Lewy body dementia, or ALS. Instead, it is used to identify assessment principles that may require adaptation to specific patterns of degeneration, motor impairment, cognitive fluctuation, and preserved or impaired communication.

The limits of assessment based on behavior are well established. Standardized neurobehavioral testing improves diagnostic accuracy, yet clinically meaningful misclassification remains common when overt responses are inconsistent or absent [3]. Functional neuroimaging has shown that some behaviorally unresponsive patients retain covert command following, first with functional magnetic resonance imaging performed during active tasks and later with more systematic approaches demonstrating willful modulation of brain activity [11,12]. Electroencephalography has extended this principle to the bedside by detecting covert awareness through motor imagery and related methods in patients who could not demonstrate command following behaviorally [13]. A subsequent reanalysis of the same dataset [14] and the corresponding author reply [15] illustrate that detection of covert command-following with EEG is sensitive to task structure, preprocessing choices, artifact handling, statistical assumptions, and convergent validation, and should not be interpreted as a simple binary output of a classifier.

These advances form the conceptual basis for brain–computer interfaces. A brain–computer interface enables communication or control via neural activity instead of peripheral motor output, making it well-suited in conditions such as amyotrophic lateral sclerosis, where consciousness may be preserved despite progressive paralysis [6,16]. Early systems based on the P300 demonstrated feasible communication in amyotrophic lateral sclerosis, including patients with severe disability, and longitudinal work showed that usable performance can persist during follow-up in selected individuals [17,18,19].

Artificial intelligence has added a layer of clinical value by enabling analysis of EEG, functional magnetic resonance imaging, and functional near-infrared spectroscopy data in a high-dimensional feature space that would be difficult to interpret visually or with conventional statistics alone [20,21,22]. Recent machine learning studies have improved prognostic stratification from routine EEG, identified residual awareness from functional magnetic resonance imaging obtained during rest, and classified prolonged disorders of consciousness from functional near-infrared spectroscopy network features [20,21,22]. Functional near-infrared spectroscopy is particularly attractive for this purpose because it is portable and suited to repeated bedside evaluation in fragile patients [23].

**Figure 1:**
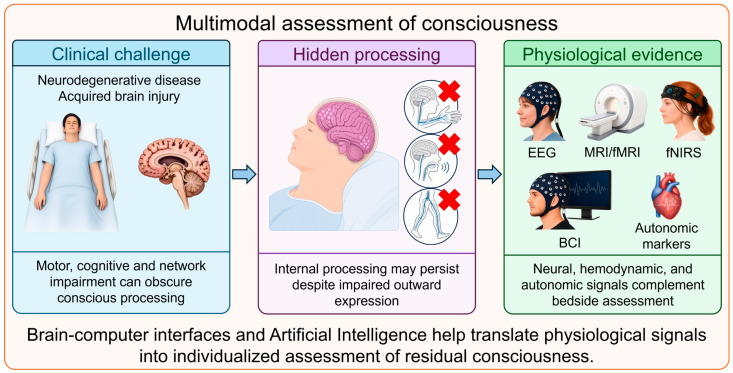
Multimodal assessment of consciousness. Neurodegenerative disease and acquired brain injury may impair motor output, cognition, and large-scale brain networks, thereby limiting the reliability of behavioral assessment. Residual internal processing may persist despite impaired outward expression. In the central panel, the red crosses indicate impaired behavioral output channels, including speech, voluntary movement, and other motor responses. EEG, MRI or fMRI, fNIRS, brain–computer interfaces, autonomic markers, and artificial intelligence provide complementary physiological evidence that may support individualized assessment of residual consciousness.

This review examines how multimodal methods developed largely in acquired disorders of consciousness, including EEG, magnetic resonance methods, functional near-infrared spectroscopy, brain–computer interfaces, and artificial intelligence, may inform future assessment of consciousness and covert cognition in neurodegenerative disease. The aim is not to equate brain injury with neurodegeneration, but to define which methodological principles may be clinically useful, which require adaptation specific to the disease, and where current evidence remains insufficient for direct clinical translation. The clinical rationale for this multimodal approach is summarized in Figure 1, which illustrates how neurodegenerative disease and acquired brain injury can create a mismatch between preserved internal processing and impaired outward expression.

### 1.1. Methodology

This article is a narrative review designed to address a clinically complex and methodologically uneven research area: the assessment of consciousness, covert cognition, and communication capacity in neurodegenerative disease when observable behavior is unreliable. The review was structured around the problem that direct evidence from neurodegenerative cohorts remains limited for several advanced assessment methods, whereas many of the most developed paradigms for detecting covert awareness, cognitive-motor dissociation, residual command-following, and impaired behavioral expression come from studies of acquired disorders of consciousness after brain injury. For this reason, the reviewed literature was organized into two levels of evidence. The first level included studies directly involving neurodegenerative diseases, especially ALS, Alzheimer’s disease, Lewy body dementia, Parkinsonian syndromes, and severe dementia. The second level included studies in acquired disorders of consciousness after brain injury, which were included when they provided methodological evidence for detecting covert awareness, cognitive-motor dissociation, residual command-following, or impaired behavioral expression. This second level was used to define transferable assessment principles, not to infer diagnostic or prognostic performance in neurodegenerative cohorts.

**Figure A1:**
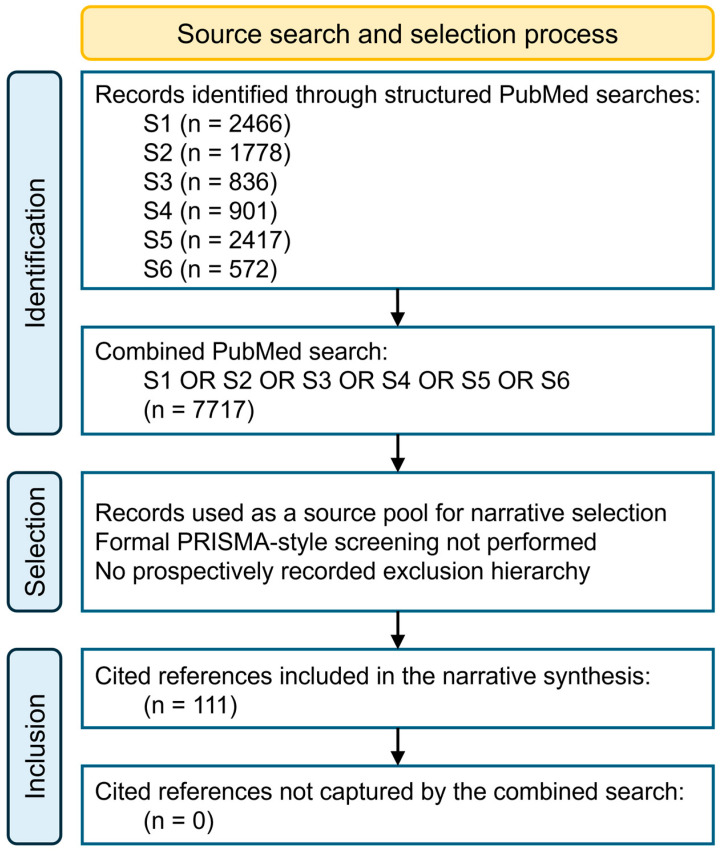
Source search and selection flow diagram. Structured PubMed title-field searches were performed in six thematic blocks. Full PubMed queries for S1–S6 are provided in Appendix A, Table A1. The final combined PubMed search yielded 7717 records. The final synthesis included 111 cited references, all captured by the final combined PubMed search.

Structured PubMed searches were performed using title-field free-text queries organized into six thematic blocks: disorders of consciousness and covert awareness; electrophysiological and neuroimaging markers of consciousness; functional near-infrared spectroscopy and bedside hemodynamic assessment; brain–computer interfaces and communication in severe motor impairment; artificial intelligence and multimodal classification or prognostication; and autonomic biomarkers, heart rate variability, baroreflex function, and vagus nerve stimulation. Searches were limited to English-language articles published from 1 January 2000 to 20 May 2026. The complete PubMed search strategy and the number of records retrieved for each thematic search block are provided in Appendix A, Table A1. Scopus and Google Scholar searches were used only as supplementary source-checking tools where appropriate. Because this article is a narrative and translational review, PRISMA and its extensions were not applied as a formal reporting standard [24]. The review did not aim to produce an exhaustive scoping map, did not use protocolized duplicate screening, and did not record a hierarchical list of exclusion reasons for all retrieved PubMed records. Instead, the PubMed search was used to define a transparent and reproducible source pool for narrative synthesis. To improve search transparency, a modified PRISMA-style search diagram is provided in Appendix A, Figure A1.

During synthesis, each study was considered according to both its thematic domain and its evidence level. Direct neurodegenerative studies were used to support statements about disease-specific assessment challenges, clinical feasibility, and translational relevance in neurodegenerative cohorts. Studies from acquired disorders of consciousness were used to support methodological principles and to identify tools that may require separate validation before clinical application in neurodegenerative disease. This distinction was used to avoid direct extrapolation from acquired brain injury to neurodegenerative disease and to identify areas where AI models, BCI paradigms, autonomic markers, and multimodal protocols require disease-specific validation.

Because the reviewed literature spans heterogeneous populations, methods, and clinical aims, the synthesis is interpretive and not quantitative. The review does not attempt to rank modalities by diagnostic superiority. Instead, it examines how electrophysiology, neuroimaging, fNIRS, BCI systems, artificial intelligence, and autonomic biomarkers may provide complementary information about consciousness, covert cognition, communication potential, and prognosis when behavior alone is an incomplete clinical measure.

To strengthen the critical component of the review, a targeted appraisal was performed for selected diagnostic, prognostic, and AI studies that were central to the clinical arguments of the manuscript (Appendix A, Table A2). Because the reviewed literature includes heterogeneous study designs, a single formal appraisal tool was not suitable for uniform application across all cited sources. Instead, the appraisal focused on domains relevant to diagnostic and prognostic translation: patient selection, clinical reference standard or comparator, sample size and cohort heterogeneity, external validation, interpretability, feasibility of bedside or real-world use, and risk of generalization issues across etiologies. This appraisal was used to distinguish methodological proof of principle from evidence closer to clinical implementation.

For a narrower subset of diagnostic and diagnostic-translational studies, especially acquired-DoC studies used in this review as methodological evidence rather than as directly transferable clinical evidence for neurodegenerative disease, a QUADAS-2-based structured assessment was additionally performed (Appendix A, Table A3). This assessment was used to make transparent the main risk-of-bias and applicability issues related to patient selection, index-test conduct, reference standard, and flow/timing, while recognizing that some proof-of-principle, prognostic, reanalysis, and machine-learning studies only partially fit the conventional QUADAS-2 structure [25].

The PRISMA 2020 and QUADAS-2 references were added as methodological citations and were not counted as database-search records or as included studies in the literature flow.

### 1.2. Conceptual Scope: From Acquired Brain Injury to Neurodegenerative Disease

A central distinction in this review is between the source of the strongest current evidence and the clinical field in which these methods may require future adaptation. Acquired disorders of consciousness after traumatic, hypoxic–ischemic, vascular, or other acquired brain injury have provided the most mature evidence for detecting covert awareness, cognitive-motor dissociation, command-following, and residual cognitive processing without reliable behavioral output. These studies have shaped active fMRI paradigms [11,12], command-following tasks monitored with EEG [13], perturbational and connectivity measures [26,27,28,29,30,31,32], fNIRS protocols [33,34,35,36,37], and BCI approaches [38,39].

Neurodegenerative diseases differ clinically and physiologically from acquired brain injury. In ALS and related motor neuron disorders, cognition and awareness may remain relatively preserved while motor and bulbar output progressively fail [17,18,40,41,42,43]. In Alzheimer’s disease, Lewy body dementia, Parkinsonian syndromes, and other dementias, impaired awareness may coexist with progressive cognitive decline, attentional fluctuation, impaired language, apraxia, sensory impairment, sleep–wake disturbance, medication effects, and variable cooperation [4,5,7,8,9,10,44,45,46,47]. These differences affect not only clinical interpretation but also the generalizability of AI models, because models trained on acquired brain injury cohorts may learn patterns of structural damage, arousal disturbance, network disconnection, medication exposure, or recovery trajectory that are not equivalent to those seen in neurodegeneration [48,49,50,51,52,53,54,55,56,57].

For this reason, the present review does not treat evidence from acquired brain injury as directly transferable to neurodegenerative disease. Instead, acquired brain injury is used as the methodological field in which multimodal consciousness assessment has been most extensively developed. Neurodegenerative disease is treated as a translational field in which these methods may be useful but require adaptation, validation, and interpretation according to mechanisms specific to each disease, clinical trajectories, and patterns of preserved or impaired communication. To make this translational logic explicit, Table 1 separates the main clinical contexts reviewed in this article, the assessment problem addressed in each context, and the expected degree of transferability to neurodegenerative disease.

**Figure 2:**
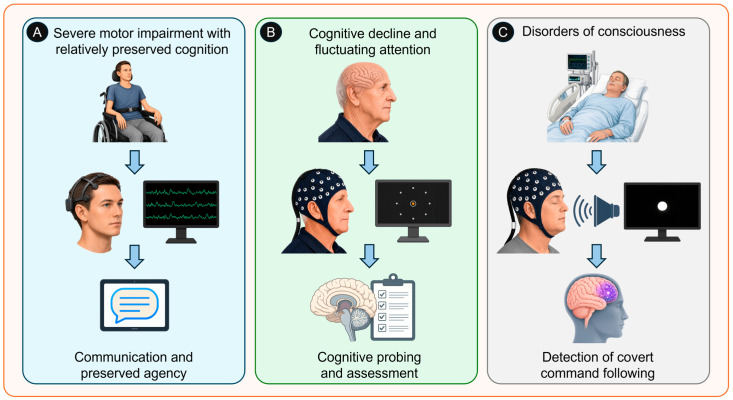
Clinical contexts in which behavioral assessment may underestimate preserved cognition, awareness, or communication potential across distinct etiologies. (**A**) In severe motor impairment with relatively preserved cognition, as in advanced motor neuron disease or locked-in states, brain–computer interface systems may provide a pathway from neural activity to communication and preserved agency when conventional motor output is lost. (**B**) In neurodegenerative conditions associated with cognitive decline and fluctuating attention, electrophysiological or BCI-based paradigms may support structured cognitive probing when verbal cooperation, sustained attention, or motor responses are unreliable. (**C**) In disorders of consciousness, active EEG, fNIRS, fMRI, or BCI paradigms may help detect covert command-following and residual awareness that are not evident during bedside examination. Together, these scenarios illustrate why multimodal assessment is needed when observable behavior is an incomplete measure of consciousness. The figure does not imply mechanistic equivalence between acquired brain injury and neurodegenerative disease. Rather, it illustrates shared assessment problems that may justify cautious methodological transfer between fields.

Figure 2 illustrates three situations in which behavior alone may fail to capture preserved cognition, fluctuating awareness, or covert command-following.

## 2. Electrophysiological and Neuroimaging Techniques in Consciousness Assessment

Electroencephalography remains one of the most practical neurophysiological tools for assessing consciousness because it combines millisecond temporal resolution with bedside feasibility and can capture neural responses on the timescale of conscious access and state fluctuations. Most of the evidence summarized in this section comes from acquired disorders of consciousness and should therefore be read as a methodological foundation for neurodegenerative applications and not as validation specific to the disease. Event-related potential studies have shown that violations of auditory regularity can reveal residual hierarchical processing even in severely impaired patients. In one report, violations of local and global auditory structure elicited differential responses across diagnostic groups, with the global effect being more closely related to preserved consciousness than the automatic detection of local novelty [58]. Single-trial decoding of the same local–global auditory design showed that late responses to global novelty can distinguish minimally conscious from more deeply impaired patients, supporting EEG as a tool for detecting covert cognition beyond the bedside examination [59]. A large multicenter screening study then found that several EEG markers, including low-frequency power, complexity, and long-range information exchange, can discriminate vegetative or unresponsive states from minimally conscious states, with partially independent diagnostic value across markers [60]. Together, these findings established that EEG is more than a monitor of background slowing and can provide a quantitative approach to residual conscious processing. Resting EEG has also revealed state differences in the organization of networks distributed across the brain. Spectral network analysis showed that disorders of consciousness are associated with progressive disruption of connectivity in the alpha band and with reorganization toward a less integrated and less complex network structure as consciousness declines [61]. In that study, some behaviorally unresponsive patients with covert awareness identified by other techniques showed relatively preserved alpha networks, which implies that EEG features derived from network organization may more accurately reflect latent consciousness than behavior alone [61]. High-density EEG combined with direct cortical perturbation has provided an additional approach via the perturbational complexity index. This index was developed to quantify the integrated and differentiated character of cortical responses after transcranial magnetic stimulation. It was shown to distinguish conscious from unconscious conditions independently of sensory input and motor behavior [26]. Subsequent work in unresponsive patients found that the same index could stratify patients by the likelihood of residual consciousness and identify a subgroup of behaviorally unresponsive individuals with complex cortical responses compatible with preserved conscious capacity [27].

Magnetic resonance imaging contributes a different level of information because it characterizes the structural substrate on which conscious processing depends. Diffusion tensor imaging and diffusion MRI-derived measures have distinguished vegetative or unresponsive states from minimally conscious states by demonstrating more severe abnormalities in subcortical white matter and thalamic regions in the former group, which points to impaired structural connectivity as a major determinant of clinical unresponsiveness [66]. Structural disconnection involving pathways that support overt responsiveness has remained particularly important because preserved behavioral responsiveness depends on the integrity of fibre tracts involved in motor output, including connections between the thalamus and primary motor cortex and related cerebellar motor pathways [67].

Functional MRI has shown that consciousness is closely related to the integrity of intrinsic brain networks, and not to activity in a single region. Resting-state studies found that default mode network connectivity decreases in proportion to the degree of consciousness impairment across coma, vegetative states, minimally conscious states, and locked-in syndrome, establishing network connectivity as a graded marker, not a binary sign [28]. Further work indicated that the default mode network is biologically relevant to the structural basis of disordered consciousness, because disrupted connectivity within this system was linked to the neural substrate of impaired consciousness [29]. Effective connectivity analyses refined this view by showing altered directional interactions centered on the posterior cingulate cortex, which supports the interpretation that impaired self-related and integrative network dynamics may be involved in reduced consciousness [68]. At the level of the individual patient, intrinsic functional connectivity has differentiated minimally conscious from unresponsive patients with high discriminative performance, with auditory and long-range multisensory networks emerging as particularly informative [30]. Posteromedial functional connectivity patterns have also predicted consciousness level and later recovery outcomes in acquired brain injury, underscoring the prognostic value of magnetic resonance network analysis [31]. Dynamic resting-state analyses extended these observations by showing abnormal dwell-time and transition properties of functional connectivity states in disorders of consciousness, which favors the view that impaired consciousness also involves reduced flexibility in large-scale network reconfiguration [32].

Functional near-infrared spectroscopy has emerged as a complementary method with strong translational promise. Its principal advantage is portability, which enables repeated bedside evaluation in patients who cannot be transported easily for MRI. Resting-state fNIRS studies have identified disrupted prefrontal network topology in disorders of consciousness and have shown that specific connections involving Brodmann area 10 can distinguish minimally conscious from unresponsive patients with high classification performance [35]. Task-based fNIRS has also detected residual awareness through motor imagery tasks performed in response to commands. In one cohort, patients in a minimally conscious state showed significant hemodynamic responses during active tasks, whereas patients in an unresponsive state did not, and the hemodynamic measures correlated with Coma Recovery Scale Revised scores in the minimally conscious group [34]. More recently, bedside fNIRS has been shown, in a small acute severe brain injury cohort, to detect preserved consciousness that may not be evident from bedside behavior alone [36].

Across modalities, convergence is the dominant trend. EEG offers temporal precision and direct access to neural dynamics. MRI and fMRI define structural disconnection and large-scale functional architecture. fNIRS provides a portable hemodynamic window that can be repeatedly deployed at the bedside. The clinical challenge is no longer the absence of measurable brain signals, but the integration of these signals into an interpretive model that can distinguish absent or fluctuating consciousness, and covert but behaviorally inaccessible awareness. This multimodal perspective is important in neurodegenerative diseases, where motor impairment, cognitive fluctuations, and selective network degeneration can distort purely behavioral assessment and provide a strong rationale for combining electrophysiological and neuroimaging measures in the evaluation of consciousness. However, these methods should be regarded as translational candidates for neurodegenerative disease, and not as specific standards until they are validated in cohorts defined by neurodegenerative etiology, disease stage, cognitive profile, and communication capacity.

The main modalities discussed in this section differ in signal type, clinical feasibility, and vulnerability to confounding. Table 2 summarizes their principal readouts, translational advantages, and practical limitations.

## 3. Brain–Computer Interfaces Across Acquired Brain Injury and Neurodegenerative Disease

Brain–computer interfaces occupy a bridge position between acquired disorders of consciousness and neurodegenerative disease because they can test covert command-following when behavior is absent and support communication when motor output progressively fails. Within neurodegenerative disease, the most direct evidence comes from ALS and related severe motor impairment. Amyotrophic lateral sclerosis is the clearest example because communication becomes increasingly constrained as bulbar and limb function decline. At the same time, a subset of patients retains sufficient task engagement to use neural signals for external control [40]. Early P300 systems showed that paralyzed participants could use event-related responses to select commands on a screen, although performance was heterogeneous and depended on sustained attention and visual processing [78]. Later studies in ALS demonstrated that a sizeable subset of severely disabled patients could complete BCI evaluation and achieve control that was sufficient for communication, which supported the clinical feasibility of this approach in a clinically relevant ALS sample [40]. Electrophysiological analysis also showed that people with severe ALS could achieve performance based on the P300 comparable to that of age-matched controls despite differences in ERP morphology associated with the disease, suggesting that classifier design may need to be adapted even when behavioral output remains usable [63].

The greatest translational value of BCI in ALS lies in preserving communication as disability progresses. Longitudinal home studies demonstrated that EEG systems can be deployed outside the laboratory and used independently for months by some patients with advanced ALS, with a technical complexity acceptable relative to the perceived benefit [41]. These results are clinically important because the gap between initial demonstration of feasibility and real-world assistive use has historically limited the impact of neurotechnology on severe paralysis. Implanted systems have pushed this principle further. A fully implanted cortical BCI enabled autonomous home communication in a locked-in patient with late-stage ALS, with reliable typing based on attempted movement and not on residual eye control [62]. These studies indicate that BCI in ALS is a practical means of preserving agency when conventional communication breaks down [41,43].

In dementia, the role of BCI is less mature but still important. Direct communication restoration is usually not the primary goal because motor paralysis is not the dominant impairment in most patients. Instead, BCI is being explored to detect cognitive decline, track attentional engagement, and develop assessment tools that do not depend on motor output. P300 measures have shown sensitivity to cognitive deterioration in Alzheimer’s disease, with abnormalities linked to impaired language, memory, and executive function [44]. Similar work in mild cognitive impairment and Alzheimer’s dementia found that visual oddball P300 measures can complement neuropsychological testing and may help identify clinically meaningful cognitive dysfunction when standard bedside interaction is incomplete or otherwise limited [45].

This line of work remains preliminary, yet it points toward an important conceptual extension. In neurodegenerative disease, BCI may serve as a means of communication when conventional expression is impaired, and as a structured probe of attention, target selection, and goal maintenance. That is important in disorders with cognitive fluctuation, impaired initiation, or reduced verbal cooperation. Initial neurofeedback studies using BCI training based on the P300 have shown short-term enhancement of ERP features associated with attention and improved behavioral performance after training, providing a mechanistic basis for future cognitive rehabilitation approaches in populations at risk of attention decline. Although such studies were not conducted in dementia cohorts, they support the idea that BCI systems can shift from passive recording to interactive neurorehabilitation [79].

BCI has also gained importance in the assessment of minimal consciousness and related states, where the central problem is not degeneration alone but the inability to translate preserved cognition into reliable behavior. This has direct relevance to neurodegenerative disease because severe motor impairment, fluctuating arousal, and network dysfunction can all produce a mismatch between inner state and clinical examination. Conceptual work in minimally conscious patients suggested that repeated BCI interaction might help preserve or reveal thinking directed toward a goal by restoring a contingency between covert intention and an external consequence [80]. A hybrid visual BCI combining P300 and steady-state visual evoked responses later detected awareness in a subset of patients with disorders of consciousness, including individuals without dependable bedside evidence of command following [39]. Another study showed that an auditory P3-based BCI could identify the ability to follow commands in one minimally conscious patient who lacked a behavioral response at the bedside, illustrating the diagnostic value of BCI when standard bedside examination underestimates preserved cognition [38].

Subsequent work expanded assessment based on BCI and EEG beyond simple detection of the ability to follow commands. A hybrid system combining P300 and steady-state visual evoked responses identified number processing and mental calculation in patients with disorders of consciousness, implying that some patients could engage in covert cognitive operations of a higher order, and not only orienting responses [81]. EEG studies of covert cognition also showed that active paradigms can reveal the ability to follow commands in patients whose bedside profile suggests more severe impairment, while simultaneously highlighting the variability and fragility of these signals across repeated testing [82]. More recent audiovisual P300 studies found that BCI accuracy correlates with Coma Recovery Scale Revised scores and with later clinical improvement, which favors the interpretation that BCI metrics may carry prognostic as well as diagnostic information [83].

Several future directions emerge from this body of work. First, BCI systems need to become more tolerant of sensory loss, fatigue, and limited learning capacity. This is highly relevant in advanced neurodegeneration, where visual dysfunction, slowed processing, and fluctuating alertness may reduce the utility of conventional visual spellers. Tactile and simplified paradigms, therefore, represent an important direction. A recent P300 approach combining electrical and vibration stimulation was specifically designed for users with lower learning ability or difficulty sustaining attention, and it demonstrated promising performance in classifying commands without relying on complex visual search [84]. Second, deployment in the home and remote calibration are likely to become standard design goals instead of optional refinements, because long-term usefulness depends more on daily reliability than on peak laboratory accuracy [41]. Third, implanted and hybrid systems may increase the range of patients who can benefit when ocular control, scalp signal quality, or prolonged attention are insufficient for noninvasive platforms [39,43].

Overall, BCI represents the strongest direct bridge between consciousness assessment and neurodegenerative disease, particularly in ALS, where the core clinical problem is not the absence of awareness but the loss of behavioral channels through which awareness and intention can be expressed [40,41]. In minimally conscious states, BCI provides a route to detect covert command following and hidden cognitive capacity that would otherwise remain clinically invisible [38,82]. These combined roles make BCI an important component of multimodal assessment strategies for conditions in which behavior alone is an incomplete index of consciousness and cognition.

## 4. AI-Assisted Multimodal Integration, Validation, and Generalization

### 4.1. Evidence from EEG, fMRI, fNIRS, and Multimodal Models

The importance of artificial intelligence in this field stems from the limitations of conventional clinical and visual assessment. Modern imaging and neurophysiological testing techniques provide multidimensional, high-resolution, and complex data that are beyond the scope of interpretation based on manual inspection. This view, in the case of disorders of consciousness, has received support in the literature [48,49]. Machine learning and deep learning are more than tools for automation, but become methods for extracting latent structure from signals specific to modality. These include multimodal FDG-PET and EEG features, which have been integrated to improve diagnosis and prognostication [76]; resting-state functional magnetic resonance imaging (rsfMRI) combined with laboratory parameters has supported outcome prediction [77]; and fNIRS-based classifiers have been used to detect residual awareness or cognitive-motor dissociation at the bedside [33,37]. These approaches are clinically relevant because they may help model neuroimaging and neurophysiological data as patient-level indicators of preserved awareness, covert cognition, or recovery potential and not just as isolated group-level abnormalities [21,76,77]. Review work has emphasized that AI-based approaches may support diagnosis, prognosis, and differentiation between unresponsive wakefulness syndrome and minimally conscious states. Meanwhile, they require standardized data protocols, interpretability, and validation across heterogeneous clinical cohorts [48,49]. This matters in neurodegenerative disease because consciousness-related dysfunction often coexists with cognitive and behavioral changes, which can obscure biologically meaningful signals during routine clinical assessment. Analysis based on artificial intelligence, thus, offers a route from descriptive neuroimaging toward patient-level inference, if models are externally validated and clinically interpretable.

In EEG research, AI has expanded the range of usable markers beyond standard spectral slowing. Machine learning classifiers have identified discriminative EEG responses to familiar and non-familiar emotional videos in patients with disorders of consciousness and in healthy controls, with classification patterns that were generally consistent with subsequent clinical evolution in most patients [85]. More recent work using approximate entropy-derived EEG topographic maps and deep learning demonstrated that automated models could distinguish minimally conscious states from vegetative or unresponsive wakefulness syndrome with substantial accuracy, which provides evidence that hidden nonlinear properties of the EEG signal carry clinically useful information not captured by conventional inspection alone [86]. Another study identified five dynamic EEG connectivity states whose occurrence probability closely tracked consciousness level across the spectrum from coma to conscious control participants, and these state probabilities also predicted recovery at the individual level. These observations indicate that AI does not only compress EEG data into a diagnostic label. It can identify temporally evolving brain states that may be more biologically informative than static averages [87]. A large multicenter Chinese cohort strengthened this translational direction by showing that a combined clinical-EEG machine learning model outperformed single-modality models for classifying consciousness levels, with SHAP analysis identifying the features that contributed most to the model output and thereby improving interpretability [50].

AI has also improved the analysis of magnetic resonance data. Resting-state functional magnetic resonance imaging provides rich information about the integrity of large-scale brain networks, but its high dimensionality and interindividual variability make simple interpretation based on thresholds difficult. A multi-domain prognostic model that combined rsfMRI with laboratory data improved the prediction of outcomes in disorders of consciousness, demonstrating the value of combining imaging and non-imaging variables within a single predictive model [77]. More recent deep learning work showed that rsfMRI can help detect neural signatures compatible with covert awareness in patients with minimally conscious states and cognitive-motor dissociation, supporting the view that AI can extract diagnostically meaningful representations even when overt behavior is limited [21]. Personalized computational modeling has extended this approach by showing that connectivity features and local regional parameters contribute differently to diagnosis and prognosis. That highlights a move toward individualized modeling of consciousness assessment instead of a uniform model applied to all patients [52]. Another multimodal neuroprognostic model integrated behavioral assessment, hormonal measures related to the pituitary gland, and rsfMRI, showing that static and dynamic functional connectivity, CRS-R motor scores, and free triiodothyronine contributed complementary information for predicting prognosis in chronic disorders of consciousness [53].

Multimodal AI frameworks are important because no single modality captures the full biological substrate of consciousness. Multimodal FDG-PET and EEG analysis improved both diagnosis and prognostication relative to either modality alone. It may be regarded as an early demonstration that integrated models can outperform isolated biomarkers [76]. A subsequent intensive care cohort study showed that multimodal prediction combining electrophysiology, imaging, and clinical information could estimate 3-month and 12-month outcomes after acute disorders of consciousness. This again supports the principle that integrated models are more clinically informative than approaches specific to modality [54]. The same principle is reflected in more recent multicenter machine learning work that integrates structural imaging, resting-state functional imaging, and neurophysiological measures to improve diagnostic and prognostic stratification across heterogeneous patients with disorders of consciousness [51]. Explainable machine learning models add clinical value by indicating which clinical or EEG features drive classification, thereby reducing the black-box character of automated assessment and supporting patient-level interpretation [50]. In neurodegenerative disease, such integration is likely to be even more important, as atrophy patterns specific to the disease, network disconnection, autonomic and endocrine disturbances, and fluctuating electrophysiological states may provide complementary information instead of redundant signals [51,53].

Functional near-infrared spectroscopy is also increasingly shaped by analysis driven by artificial intelligence. An early motor imagery study based on commands used support vector machine classification of fNIRS signals to detect residual awareness and yes-or-no responses in patients with prolonged disorders of consciousness, including accurate response decoding in one minimally conscious patient [33]. Subsequent work showed that fNIRS can evaluate residual cognition and identify hemodynamic differences associated with task performance between minimally conscious and unresponsive patients, with signal features that correlate with clinical scale scores [34]. More recently, fNIRS combined with support vector machine analysis was used to detect cognitive-motor dissociation, in which preserved intention cannot be expressed behaviorally [37]. These studies suggest that AI may increase the clinical utility of portable hemodynamic monitoring by translating weak and noisy bedside signals into probabilistic classification outputs that may support assessment of preserved cognition or awareness, but require validation against clinical and neurophysiological reference measures [33,37,65].

### 4.2. Generalization and Neurodegenerative Translation

A key limitation for AI translation is that models may not generalize across etiologies. Models trained predominantly on acquired brain injury cohorts may perform well when tested on patients with similar mechanisms of injury, time from onset, structural disconnection, medication exposure, and recovery trajectories. However, they may not generalize to neurodegenerative disease, where pathology is progressive, regionally selective, and often accompanied by chronic cognitive decline, sensory impairment, sleep–wake disruption, dysautonomia, and variable cooperation. In ALS, preserved cognition may coexist with profound motor output failure, whereas in dementia, impaired responsiveness may reflect a mixture of reduced awareness, impaired comprehension, attentional fluctuation, aphasia, apraxia, or executive dysfunction. AI models intended for neurodegenerative applications therefore require separate validation, recalibration, or transfer-learning strategies in disease-specific cohorts. External validation across etiologies should be treated as a prerequisite before model outputs are used for clinical interpretation.

A cautious interpretation of these developments is that AI may support patient-level stratification instead of immediate personalized clinical decision-making. Machine learning models have been used to estimate functional outcome in prolonged disorders of consciousness, and functional or structural connectivity studies indicate that patients with similar bedside profiles may differ in preserved network organization [88,89]. Longitudinal multimodal protocols and computational models may help describe changes in state over time [90,91]. However, these approaches remain mainly investigational. Their clinical value will depend on prospective testing, external validation, transparent model outputs, and evidence that model-guided assessment improves decisions beyond repeated behavioral examination and conventional neurophysiology.

The same logic also extends to neurodegenerative diseases in general. In Alzheimer’s disease, deep learning applied to structural magnetic resonance imaging has been evaluated as a diagnostic tool supporting decisions in hospital cohorts [55]. EEG analysis based on deep learning has also classified normal cognition, mild cognitive impairment, and dementia from resting and recordings related to tasks, which illustrates how AI can convert inexpensive electrophysiological data into clinically useful staging information [56]. Machine learning models using routine EEG have distinguished Alzheimer’s disease from frontotemporal dementia, which is relevant for differential diagnosis when clinical syndromes overlap [92]. Multimodal deep learning has also been used to predict trajectories of cognitive decline in Alzheimer’s disease research settings, thereby supporting patient-level prognostic stratification and trial design [57]. These studies do not measure consciousness directly, but they show how AI-based neuroimaging and neurophysiology can support more detailed phenotyping in neurodegeneration and complement assessments oriented around consciousness when cognitive decline and reduced responsiveness intersect. Overall, AI-assisted analysis should be viewed as a tool for structured integration of multimodal data and not as a clinically established system for personalized consciousness assessment. The reviewed studies support the feasibility of patient-level classification, prognostic modeling, and multimodal feature integration, but they also highlight the need for external validation, interpretability, harmonized preprocessing, and testing across etiologies. In the context of neurodegenerative disease, AI models should therefore be considered promising translational tools, not independent agents.

### 4.3. Feature Robustness, Multimodal Fusion, Interpretability, and Bedside Implementation

The robustness of analysis supported by AI in consciousness assessment depends not only on classification accuracy, but also on the stability and interpretability of the features used as model inputs. In EEG studies, responses to events can capture residual auditory processing and violations of expected stimulus regularities, whereas spectral power, low-frequency activity, alpha band organization, connectivity measures, and complexity indices can describe background network organization and residual cortical integration [20,58,59,60,61]. More recent computational studies have extended this approach by using stimulus response patterns, nonlinear EEG features, topographic maps calculated from entropy measures, and dynamic EEG states, suggesting that information relevant to consciousness may be distributed across frequency bands, scalp regions, and changing connectivity patterns, not contained in one isolated marker [85,86,87]. Spectral, connectivity, and nonlinear EEG features have been used to distinguish levels of consciousness in patients with DoC, including large-scale EEG screening and analyses of reorganized spectral networks [60,61]. However, spectral features are vulnerable to sedation, state of sleep or wakefulness, artifacts, and nonspecific encephalopathy, whereas connectivity and nonlinear measures depend strongly on preprocessing, reference montage, recording length, and artifact control. Feature robustness should therefore be evaluated across cohorts, devices, and clinical states, not only within one development dataset, as emphasized in recent discussions of AI translation in DoC [48,49].

For fNIRS, the most relevant features include oxyhemoglobin and deoxyhemoglobin responses evoked by tasks, hemodynamic patterns recorded at the channel level during motor imagery or command following, and resting connectivity or graph measures of network organization [22,23,33,34,35,36,37]. Although fNIRS is portable and repeatable, its signals are influenced by extracerebral blood flow, optode placement, systemic physiology, hair and scalp properties, and limited cortical depth, so they should not be interpreted as direct equivalents of neural activation without appropriate preprocessing and physiological controls [23,65]. Active EEG and fNIRS paradigms may provide stronger evidence of intentionality compared with passive recordings, but they also carry a higher risk of false negative results when patients cannot understand instructions, sustain attention, perceive the stimulus, or maintain stable arousal [13,33,37,82].

Multimodal AI models can combine heterogeneous data in several ways. In early fusion, features from different modalities are concatenated before model training, allowing the classifier to learn interactions between modalities but increasing vulnerability to missing data, scale imbalance, and overfitting. In late fusion, separate models are trained for each modality and their outputs are combined at the decision level, which may be more robust when modalities differ in temporal resolution, spatial resolution, signal quality, and availability. Intermediate fusion uses representations learned from each modality and combines them in a shared latent space. This approach is conceptually attractive for EEG, fNIRS, fMRI, FDG-PET, clinical scores, and laboratory variables, but it requires larger datasets than are usually available in DoC or advanced neurodegenerative disease. Empirically, multimodal FDG-PET and EEG improved diagnosis and prognostication in DoC [76]. Models combining rsfMRI with laboratory parameters supported outcome prediction [77], whereas multimodal prediction in acute DoC has integrated electrophysiological, imaging, and clinical information [54]. More recent multicentre work supports the value of combining structural imaging, resting-state functional imaging, and neurophysiological measures [51].

Feature importance can support clinical trust only when it can be translated into neurophysiological terms. For EEG, relevant explanations may include scalp topographies, contributions of frequency bands, time windows of responses to events, connectivity edges, or probabilities of dynamic states. For fNIRS, interpretable outputs may include channel maps, hemodynamic curves during tasks, and network measures anchored to approximate cortical regions. For fMRI, clinically meaningful explanations should refer to established systems such as the default mode network, thalamocortical connectivity, auditory or multisensory networks, and motor output pathways. Post hoc explanation methods such as SHAP values, permutation importance, or saliency maps are useful only when they identify physiologically plausible features, not recording artifacts, site effects, medication exposure, acquisition protocol, or other confounders [48,49,50].

A distinction is required between offline classification and online bedside operation. Many AI models in this field are trained and evaluated offline, after artifact correction, feature extraction, parameter tuning, and cross-validation. This setting is important for biomarker discovery and retrospective prognosis, but it does not guarantee that the model can support assessment in real time or BCI operation. Online BCI systems require low latency, stable signal quality, rapid artifact rejection, adaptive calibration, and feedback that is fast enough to preserve the link between intention and consequence. This is particularly important in patients with ALS, advanced dementia, or disorders of consciousness, where fatigue, slow processing, sensory impairment, fluctuating vigilance, and limited training tolerance may reduce performance during the session [18,40,41,42,43].

The transition from laboratory recordings to bedside or home use introduces additional constraints. General BCI work has long emphasized the importance of signal acquisition, calibration, and user-specific control [6,16]. EEG with high electrode density and wet electrodes can provide richer spatial information, but it is time-consuming and difficult to deploy repeatedly in fragile patients. Systems with low electrode density, dry electrodes, or a wearable design are more practical, but they may have a lower signal-to-noise ratio, higher impedance, fewer spatial features, and greater sensitivity to movement and environmental artifacts. These issues become especially important during home deployment, where daily reliability and setup burden may determine whether the system is clinically useful [41]. Hybrid BCI approaches may partly improve robustness by combining neural signals with additional physiological information [93]. However, such approaches do not remove the need for prospective validation using the same hardware, montage, acquisition environment, and clinical workflow intended for real-world use.

Finally, progress is limited by the small data problem. Disorders of consciousness, advanced ALS, and severe neurodegenerative states are difficult to study in large, standardized cohorts, and datasets often differ in etiology, time from injury or diagnosis, medication exposure, acquisition protocol, behavioral scale, preprocessing pipeline, and outcome definition [48,49,51]. Transfer learning, data augmentation, synthetic data generation, learning from unlabeled data, and federated learning may reduce this limitation, but each introduces risks. Synthetic data may reproduce artifacts or unrealistic distributions; transfer learning may transfer bias from the source domain; and federated learning still requires harmonized labels, preprocessing, and governance [49,50,51]. Therefore, technical innovation should be paired with multicenter validation, transparent reporting, and open or shared benchmark datasets whenever feasible. In neurodegenerative disease, AI should therefore be treated as a candidate tool for multimodal integration and stratification, not as an independent clinical arbiter of awareness or prognosis.

## 5. Autonomic Measures as Supportive Physiological Context

Autonomic measures are not direct markers of consciousness, but they may provide auxiliary physiological context for interpreting arousal and engagement during multimodal assessment. Heart rate variability, indices based on entropy, and baroreflex sensitivity reflect cardiovascular regulation and its interaction with central autonomic networks, but they are strongly influenced by cardiovascular disease, medication, circadian variation, and recording conditions.

In disorders of consciousness, several studies suggest that heart rate variability may carry information related to arousal regulation and clinical state. HRV measures have differed between unresponsive wakefulness syndrome and minimally conscious state, and the addition of HRV descriptors to EEG features has improved classification in selected cohorts [72]. Indices of HRV complexity have also been associated with CRS-R scores and with resting-state fMRI connectivity within the central autonomic network [69]. These findings support the view that autonomic activity may index residual central regulation or physiological engagement. However, the interpretation remains indirect. A change in HRV may reflect preserved network integrity, emotional or sensory responsiveness, cardiovascular instability, medication effects, nonspecific arousal, or recording conditions instead of subjective awareness.

The temporal structure of autonomic signals may also be relevant. Electrocardiographic monitoring over 24 h has shown that patients with disorders of consciousness can retain day–night variation in heart rate and selected HRV measures, although these rhythms may be altered and less differentiated across diagnostic groups [73]. Such observations are clinically useful because they caution against interpreting a single brief recording as representative of the patient’s overall state. Repeated autonomic sampling may help identify periods of greater physiological stability or arousal, during which active EEG, fNIRS, or BCI paradigms may be more interpretable. This does not make autonomic monitoring a consciousness test. Instead, it may help define the conditions under which other tests should be performed.

Autonomic responsiveness to stimulation has been explored as another indirect index of physiological engagement. HRV responses to affective video stimuli, and auditory stimulation have been associated with changes in HRV or autonomic activation in some studies of severe brain injury and disorders of consciousness [94,95,96]. These observations are relevant because they suggest that autonomic recordings may capture physiological responsiveness to stimulation when overt behavior is minimal.

Autonomic measures have also been explored as prognostic markers in disorders of consciousness. HRV features, entropy during stimulation, and time from injury have been used in machine learning models to predict short-term outcome, and longitudinal HRV trajectories have been associated with later recovery in selected cohorts [70,71]. These studies are promising, but their clinical meaning remains limited by cohort size, heterogeneity, and the need for independent validation.

The relevance of autonomic measures to neurodegenerative disease is mainly contextual. In mild cognitive impairment, Alzheimer’s disease, Lewy body dementia, Parkinson’s disease, and population cohorts, HRV, baroreflex sensitivity, orthostatic hypotension, and related autonomic abnormalities have been associated with cognition, cognitive decline, differential diagnostic profiles, or dementia risk [46,47,64,74,75,97]. Reduced baroreflex function has also been reported in Alzheimer’s disease relative to mild cognitive impairment and healthy control participants, while population-level data link baroreflex sensitivity with long-term dementia risk in older adults [75,98]. These findings do not establish autonomic measures as biomarkers of consciousness. They indicate instead that autonomic dysfunction may shape the clinical context in which cognition, attention, communication, and responsiveness are assessed. Baroreflex sensitivity is particularly relevant here because it can indicate how cardiovascular regulation responds to changes in blood pressure, but impaired baroreflex function may also reflect vascular aging, peripheral autonomic failure, or medication effects instead of changes in awareness itself. This is particularly important when orthostatic instability, fluctuating alertness, sleep–wake disruption, medication exposure, or dysautonomia may reduce the reliability of behavioral testing or active BCI paradigms.

Autonomic measures may also be relevant to hybrid and longitudinal assessment model strategies. Hybrid BCI approaches show that physiological signals can be combined with brain signals to improve robustness and adapt system behavior [93]. More generally, combined EEG, ECG, and photoplethysmographic approaches may support long-term, time-varying assessment of cortical and autonomic activity [99]. In practical terms, HRV and baroreflex measures could help identify optimal assessment windows, quantify physiological engagement during BCI tasks, and provide auxiliary physiological context when motor imagery or evoked cortical responses are unstable. This integration may be of high importance in neurodegenerative disease, where dysautonomia and motor impairment may coexist with cognitive fluctuation and reduced communicative capacity.

In the context of this review, autonomic measures should therefore be interpreted as auxiliary markers of arousal regulation, physiological engagement, or assessment timing, not as independent evidence of awareness. Their role in neurodegenerative disease remains supportive and requires validation alongside EEG, fNIRS, BCI performance, behavioral assessment, and disease-specific clinical variables. Because the reviewed literature spans several modalities and clinical populations, Table 3 provides an evidence map.

## 6. Future Directions

**Figure 3:**
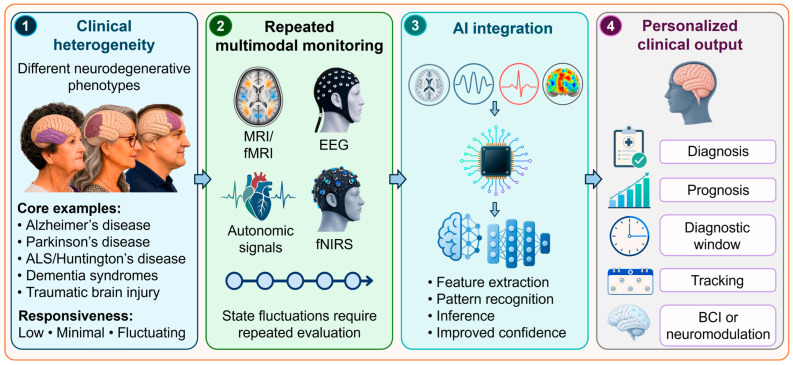
Future model of multimodal and AI-assisted assessment of consciousness and cognition. The numbered labels (1)–(4) indicate consecutive steps in the proposed assessment pathway, from clinical heterogeneity, through repeated multimodal monitoring and AI-assisted integration, to personalized clinical output. These clinical factors include fluctuating responsiveness, impaired motor output, cognitive decline, and disorders of consciousness create a need for repeated multimodal monitoring. Signals derived from MRI or fMRI, EEG, fNIRS, and BCI paradigms may be integrated using artificial intelligence, while autonomic measures may provide auxiliary physiological context to support individualized diagnostic, prognostic, and clinical interpretation, future study design, and cautious planning of therapeutic trials. The intended output is not a single universal biomarker, but a structured patient-level profile that may guide diagnosis, recovery prediction, selection of optimal assessment windows, longitudinal tracking, BCI-based communication, and selection of candidates for future neuromodulation or rehabilitation studies.

The next translational step is to determine which methods developed in acquired disorders of consciousness can be adapted to neurodegenerative disease, and under which clinical constraints such adaptation is justified. The field is now approaching a point at which adding isolated biomarkers may be less useful than learning how to read existing signals together. Studies of the default mode network have shown that network integrity can carry clinically relevant information in disorders of consciousness [101]. Functional network measures may also contribute to prognosis when they are interpreted alongside clinical characteristics instead of being treated as detached imaging findings [102]. More recent work on default mode network abnormalities, together with reviews of functional neuroimaging, has reinforced the view that impaired consciousness should be understood as a disturbance of distributed systems, not as a failure of one measurable signal [103,104]. This perspective naturally points toward longitudinal and multimodal assessment. A patient may be awake but inattentive, physiologically unstable, fatigued, unable to produce a motor response, or briefly more responsive than at the time of examination. Repeated assessment is not a methodological luxury, but a way of separating a transient state from a reproducible clinical pattern. Multicenter work that combines modalities and evaluates diagnostic and prognostic markers is especially important for this reason [51]. Portable fNIRS may be useful in this setting precisely because it can be repeated at the bedside and incorporated into monitoring, BCI paradigms, and treatment-oriented protocols [65]. Figure 3 summarizes the practical direction proposed for future research and clinical translation. Instead of treating EEG, fMRI, fNIRS, and BCI paradigms as separate tests, the figure presents them as complementary sources of information that may be integrated computationally to support patient-level assessment, prognosis, and treatment planning, with autonomic measures considered only as optional physiological context.

Artificial intelligence will face the same test. Many models perform well in the datasets in which they were developed, but clinical relevance begins only when they remain reliable outside those conditions. EEG classifiers such as DOCTer illustrate the diagnostic promise of computational approaches based on resting-state EEG, while also showing why larger and more heterogeneous datasets are important for clinical translation [105]. Recent reviews have made a similar point. Artificial intelligence may support diagnosis and prognosis in disorders of consciousness, but only if model outputs are interpretable, externally validated, and robust to heterogeneous clinical data [49]. Large multicenter models are a necessary step in this direction, particularly when they integrate information from several modalities and do not only rely on a single recording type [50]. This issue becomes even more complex when disorders of consciousness are considered alongside severe neurodegenerative disease. Advanced dementia may make awareness difficult to assess because cognition, attention, arousal, and behavioral expression deteriorate together [10]. Late-stage motor neuron disease poses a different problem. Intention may be preserved, but the channels through which intention is expressed can disappear. This is one reason why implantable BCI systems have attracted interest in advanced ALS [62]. BCI research should now move away from short demonstrations of feasibility as the main benchmark of success. The more difficult question is whether these systems can work when patients are tired, slow to respond, visually or auditorily impaired, medically fragile, or living outside a specialist research environment. Early neurointerventional BCI work has shown that implanted systems can restore useful control in severe paralysis, but this is only the beginning of the translational problem [106]. For patients with late-stage ALS, medical usefulness will depend on reliability, comfort, ethical acceptability, and sustained function in daily life [62]. Evidence that an implantable BCI can remain functional over several years is particularly important, because it shifts attention from proof of concept to durability [42]. In advanced ALS and related conditions, BCI may have a dual role: it may support communication, but it may also offer a structured way to test for preserved intention when conventional behavioral responses have disappeared.

The same pragmatism should guide clinical implementation. More intensive monitoring is not automatically better care. A useful assessment pathway is one that improves diagnosis, prognosis, or treatment decisions without creating excessive burden for the patient or the clinical team. The finding that repeated routine EEG may sometimes be clinically competitive with continuous EEG for outcome prediction after traumatic brain injury illustrates this point well [107]. Future studies should not simply ask if EEG, fNIRS, MRI, autonomic markers, or BCI testing can detect clinically important signals. They should ask which combinations are worth using, in which patients, at which stage, and in which care environment.

This matters especially in neurodegenerative medicine, where assessment tools must be usable not only in tertiary centers, but also in outpatient clinics, hospital wards, rehabilitation units, and long-term care facilities. Therapeutic translation remains a separate but connected challenge. Vagus nerve stimulation has been explored as a possible way to modulate circuits involved in arousal in patients with prolonged disorders of consciousness [108]. Systematic review evidence suggests that this area is clinically promising, although still limited by small studies and heterogeneous protocols [109]. Randomized work on transauricular vagus nerve stimulation has added support for neuromodulatory approaches, but it has not removed the need for larger controlled studies [110]. Individual patient data meta-analysis may help clarify which patients are most likely to benefit and under what conditions stimulation should be attempted [111]. The longer-term aim should be to connect assessment and intervention directly. Physiological signals would then serve not only to describe the patient’s current state, but also to guide when stimulation should be attempted, which systems should be targeted, and how rehabilitation should be adapted over time.

The most useful progress over the next decade will probably come from building clinical pathways and not from proposing more standalone markers. Bedside examination will remain the starting point, but it should be supported, when behavioral output is unreliable, by computational EEG, targeted neuroimaging, portable hemodynamic methods, autonomic measures, and BCI testing. Artificial intelligence may eventually help organize these data into diagnostic and prognostic profiles specific to each patient. The goal, however, should not be measurement for its own sake. It should be a system in which convergent physiological evidence can be translated into decisions that are clear enough, robust enough, and practical enough to be used in real clinical care. To translate the reviewed evidence into practical clinical questions, Table 4 matches common assessment problems with modalities that may be useful under specific constraints.

## 7. Limitations

This review has several limitations. First, the evidence base is intentionally translational and not etiologically uniform. The most mature literature on covert awareness, cognitive-motor dissociation, and multimodal consciousness assessment comes from acquired disorders of consciousness after traumatic, hypoxic–ischemic, vascular, or other acquired brain injury. Although this evidence is highly relevant to the general problem of dissociation between preserved inner processing and impaired outward expression, it cannot be transferred directly to Alzheimer’s disease, Lewy body dementia, Parkinsonian syndromes, amyotrophic lateral sclerosis, or other neurodegenerative conditions. Neurodegenerative disorders differ in disease tempo, anatomical selectivity, cognitive profile, motor involvement, autonomic burden, and fluctuation of arousal. The conclusions of this review therefore concern methodological adaptation and future validation, not clinical interchangeability between brain injury and neurodegeneration. Second, the available evidence is uneven across modalities. EEG, fMRI, and BCI studies have provided an important proof of principle for detecting covert command-following or residual cognitive processing, whereas fNIRS and autonomic measures are still at an earlier stage of clinical validation in many contexts. The evidence for communication through BCI is strongest in amyotrophic lateral sclerosis. Meanwhile, the role of BCI in dementia is still mainly exploratory. It should not be assumed that a method validated in one diagnostic group will have the same performance, tolerability, or interpretability in another. Third, many studies rely on small samples, single-center cohorts, heterogeneous inclusion criteria, and variable preprocessing or classification pipelines. This is particularly important for approaches based on artificial intelligence. High performance within a development cohort may not translate into robust clinical performance across hospitals, devices, recording protocols, disease etiologies, or levels of medical complexity. External validation, transparent reporting, interpretable model outputs, and prospective testing will be necessary before such approaches can be treated as clinical decision tools. Finally, physiological signals should not be interpreted as direct equivalents of consciousness, because none of these measures alone proves subjective awareness, intentionality, or communicative competence. Their clinical value is strongest when they converge with repeated behavioral assessment, longitudinal trends, and a plausible neurobiological interpretation.

The review is also subject to limitations inherent to narrative synthesis. Only English-language publications were considered, which may introduce language bias. The literature is also vulnerable to publication bias, because positive findings, successful classification models, and technically feasible BCI or AI systems are more likely to be reported than negative or unsuccessful attempts. The included studies were heterogeneous in design, cohort selection, etiology, disease stage, acquisition protocol, preprocessing pipeline, reference standard, and outcome definition, which limited the possibility of quantitative synthesis. In addition, the field is evolving rapidly, and relevant studies from non-indexed sources, preprints, conference proceedings, or smaller technical journals may have been missed. Finally, although the selection was guided by explicit clinical and methodological relevance, some degree of author judgment is unavoidable in a narrative review. This limitation was addressed by separating evidence levels, adding an evidence map, and including a targeted critical appraisal of representative studies instead of using the review to support equivalence claims across etiologies.

## 8. Conclusions

Research in acquired disorders of consciousness has shown that behavior alone can be an insufficient proxy for awareness, when motor output, arousal, attention, or cooperation is compromised, and this principle is increasingly relevant to selected neurodegenerative conditions. The strongest direct neurodegenerative application is currently found in ALS and related severe motor impairment, whereas applications in dementia and Parkinsonian syndromes remain more exploratory and require validation in each disease context. The reviewed evidence indicates that residual awareness, intentionality, and cognitive engagement may persist in selected patients despite profoundly limited outward responsiveness. A clinically useful assessment model should therefore combine bedside examination with targeted physiological measures instead of treating these approaches as separate domains. The next step for the field is to convert multimodal evidence into reliable diagnostic and prognostic decisions that improve communication, care planning, and therapeutic selection.

## Figures and Tables

**Table 1:** Translational relevance of evidence from acquired brain injury to neurodegenerative disease.

Clinical Context	Assessment Problem	Evidence Strength	Transferability	Caveat for AI Generalization
Acquired DoC after brain injury [11,12,13,26,27,28,29,30,31,32,38,39,58,59,60,61]	Covert awareness, cognitive-motor dissociation, command-following without behavior	Strongest methodological evidence	Methodological foundation	Models may learn network specific to injury and recovery patterns
ALS/locked-in states [17,18,19,40,41,42,43,62,63]	Preserved cognition with progressive loss of motor output	Strong direct neurodegenerative evidence for BCI	High for communication-focused BCI	Requires adaptation to fatigue, ocular loss, progression, home use
Severe dementia/AD [5,10,44,45,55,56,57]	Awareness and cognition difficult to assess behaviorally	Emerging evidence	Moderate/low	Cognitive impairment may confound task comprehension and command-following
Lewy body disease/Parkinsonian syndromes [4,7,8,9,46,47,64]	Fluctuating cognition, arousal, autonomic and motor impairment	Emerging evidence	Moderate	State fluctuation and medication effects may destabilize model outputs
fNIRS/EEG/fMRI paradigms from DoC [22,23,28,29,30,31,32,33,34,35,36,37,58,59,60,61,65]	Physiological detection of residual processing	Strong in DoC, limited in ND	Requires validation	Feature distributions may shift across etiologies

Note: Evidence strength and transferability are qualitative interpretive categories used for this narrative review; they are not formal grades of evidence. DoC, disorders of consciousness; ND, neurodegenerative disease; BCI, brain–computer interface; AI, artificial intelligence.

**Table 2:** Overview of multimodal assessment methods.

Modality	Main Signal or Readout	What the Method Can Reveal	Main Clinical Advantage	Main Limitation
EEG and event-related potentials [26,27,58,59,60,61]	Event-related potentials, spectral power, connectivity, complexity, perturbational complexity	Residual auditory processing, covert cognition, preserved cortical complexity, and differences between unresponsive wakefulness syndrome and minimally conscious state	Bedside feasibility, high temporal resolution, repeatability	Sensitive to artifacts, vigilance fluctuations, medication effects, and variable task engagement
Structural MRI and diffusion imaging [66,67]	White matter integrity, thalamocortical pathways, motor-output pathways	Structural disconnection that may contribute to impaired responsiveness or inability to express preserved cognition	Defines anatomical substrate of impaired responsiveness	Indirect measure of consciousness; transport and acquisition burden
Resting-state and task-based fMRI [28,29,30,31,32]	Default mode network connectivity, multisensory network connectivity, dynamic connectivity states	Network integrity, covert awareness, prognosis, and recovery potential	High spatial resolution and whole-brain network assessment	Limited bedside accessibility; requires transport, immobility, and physiological stability
Functional near-infrared spectroscopy [23,33,34,36,37,65]	Cortical hemodynamic responses, functional connectivity, motor imagery responses	Residual awareness, residual cognition, cognitive-motor dissociation, and bedside hemodynamic responses to commands	Portable, repeatable, suitable for fragile patients and bedside testing	Limited cortical depth, extracerebral signal contamination, and lower spatial coverage than fMRI
Brain–computer interfaces [18,38,39,41,42,43,62]	P300, steady-state visual evoked potentials, motor imagery, hybrid signals, implanted cortical signals	Command-following, intentionality, communication potential, and assistive control in severe paralysis	Can convert neural activity into communication or external control	Requires attention, sensory access, calibration, training, and long-term usability
Autonomic measures [69,70,71,72,73,74,75]	Heart rate variability, entropy, circadian variation, baroreflex sensitivity	Arousal regulation, physiological engagement, autonomic instability, and possible assessment timing	Noninvasive, low burden, may provide contextual physiological information during repeated assessment	Not specific for awareness; influenced by cardiovascular disease, medication, respiration, and autonomic failure
AI-assisted multimodal analysis [21,49,50,51,76,77]	Machine learning and deep learning models integrating EEG, fMRI, FDG-PET, fNIRS, clinical, laboratory, or autonomic features	Patient-level diagnostic classification, prognostic stratification, and detection of latent signal patterns	Can integrate heterogeneous data and support individualized inference	Requires external validation, interpretability, standardized preprocessing, and clinically representative datasets

Note: The table summarizes representative uses of each modality in the reviewed literature. It should not be read as a ranking of diagnostic superiority or clinical readiness.

**Table 3:** Evidence map of selected physiological, neuroimaging, BCI, and computational approaches.

Evidence Domain	Population or Context	Main Finding	Translational Relevance
EEG markers of residual consciousness [58,59,60,61]	Patients with disorders of consciousness	EEG responses to auditory regularity violations, spectral features, network organization, and complexity measures can help distinguish levels of consciousness	Supports bedside detection of residual processing beyond behavioral examination
Perturbational complexity [26,27]	Conscious, unconscious, and behaviorally unresponsive states	Cortical responses to perturbation can distinguish conscious from unconscious conditions independently of sensory input and motor output	Provides a biologically grounded index of cortical capacity for consciousness
MRI/fMRI network integrity [28,29,30,31,32]	Disorders of consciousness and acquired brain injury	Default mode network integrity, multisensory connectivity, and dynamic connectivity properties relate to consciousness level and recovery	Supports diagnosis and prognosis through network-level assessment
fNIRS-based residual awareness and cognition [22,33,34,36,37,65]	Prolonged DoC, acute severe brain injury, bedside assessment	fNIRS can detect hemodynamic responses during motor imagery, residual cognition, residual awareness, and cognitive-motor dissociation	Offers a portable hemodynamic tool for repeated bedside assessment and potential BCI integration
BCI in severe motor impairment and ALS [18,40,41,42,43,62,63]	Amyotrophic lateral sclerosis, locked-in state, severe paralysis	Noninvasive and implanted BCI systems can support communication and control when motor output is profoundly impaired	Demonstrates the clinical value of neural communication pathways when behavior is no longer reliable
BCI in disorders of consciousness [38,39,81,82,83]	Minimally conscious state and other disorders of consciousness	BCI paradigms can detect command-following, awareness, number processing, mental calculation, and prognostically relevant P300 responses	Provides a structured way to test intentionality when bedside responses are absent or inconsistent
AI-assisted multimodal models [21,51,53,54,76,77]	Acute and chronic disorders of consciousness	Integrated models combining neuroimaging, electrophysiology, clinical and laboratory variables, or behavioral scores have improved diagnostic and prognostic performance in selected study cohorts	Supports multimodal data integration, but clinical use requires external validation, interpretability, and testing across etiologies
Autonomic measures as auxiliary physiological context [69,72,73,74,75,99,100]	Disorders of consciousness, mild cognitive impairment, AD, Lewy body dementia, PD, and population cohorts	HRV, entropy, circadian variation, baroreflex sensitivity, and brain–heart interaction have been associated with arousal regulation, diagnostic category, recovery, cognition, cognitive decline, or dementia risk in selected cohorts	Provides auxiliary physiological context for arousal, engagement, assessment timing, and autonomic confounders; does not provide independent evidence of awareness

Note: The table is an evidence map based on selected representative studies cited in the review. It is not a systematic grading of all available evidence. The terms “evidence domain” and “translational relevance” indicate potential usefulness for interpretation or future research, not validated clinical readiness. Autonomic measures are included only as auxiliary physiological context and should not be interpreted as independent markers of awareness.

**Table 4:** Matching assessment methods to clinical problems.

Clinical Problem	Most Useful Approaches	Rationale	Practical Caveats
Motor output is absent or unreliable [11,12,13,38,82]	EEG, active fMRI, fNIRS, BCI	These methods can test command-following or covert cognition without requiring overt motor behavior	Negative results may reflect fatigue, sensory impairment, poor comprehension, or unstable vigilance instead of the absence of awareness
The patient is medically fragile or difficult to transport [23,36,70,72]	EEG, fNIRS, HRV, repeated bedside autonomic monitoring	Bedside-compatible tools reduce the need for transport and allow repeated assessment over time	Lower spatial specificity than MRI; interpretation depends on signal quality and physiological context
Arousal, vigilance, or attention fluctuates [60,61,69,73]	Repeated EEG, longitudinal HRV, fNIRS, repeated behavioral assessment	Repeated measurements can separate transient state changes from reproducible clinical patterns	Single recordings may underestimate preserved capacity or overinterpret transient responses
Advanced ALS or locked-in state limits communication [18,40,41,42,43,62]	Noninvasive BCI, implanted BCI, EEG-based communication systems	Cognition and awareness may remain relatively preserved while motor and bulbar output fail	Long-term value depends on comfort, reliability, caregiver burden, calibration, and ethical acceptability
Advanced dementia reduces cooperation with standard assessment [10,44,45]	EEG, ERP, simplified BCI paradigms, fNIRS, autonomic measures	These tools may reduce dependence on verbal response, complex instruction-following, or motor output	Interpretation is complicated by cognitive impairment, attention deficits, sensory problems, and reduced task comprehension
Differential diagnosis or prognosis is uncertain [21,50,51,76,77]	Multimodal AI, rsfMRI, EEG, FDG-PET, clinical-laboratory models, autonomic measures	Integrated models may capture complementary information that no single modality provides	Requires external validation, transparent reporting, harmonized protocols, and clinically interpretable outputs
Covert cognition or cognitive-motor dissociation is suspected [12,13,21,33,37]	Active EEG paradigms, fMRI, fNIRS with classification, BCI	These approaches can identify preserved intention or task engagement when behavior is absent or inconsistent	Requires careful control of command comprehension, sensory access, and false-negative risk
Planning neuromodulation or rehabilitation [109,110,111,112,113]	EEG, HRV, fNIRS, longitudinal multimodal monitoring	Physiological measures may help describe state changes and candidate stimulation windows, but evidence remains insufficient for routine clinical selection	Evidence remains heterogeneous; optimal stimulation protocols and patient selection criteria are not yet settled
Long-term monitoring outside specialist laboratories is needed [41,42,90,99]	Home-compatible BCI, repeated EEG, wearable ECG/PPG, longitudinal multimodal protocols	Longitudinal data may be more clinically informative than isolated high-complexity testing	Usability, adherence, data quality, and clinical workflow integration become central limitations

## Data Availability

No new data were created or analyzed in this study. Data sharing is not applicable to this article.

## Abbreviations

The following abbreviations are used in this manuscript:

AD	Alzheimer’s disease
AI	artificial intelligence
ALS	amyotrophic lateral sclerosis
AUC	area under the curve
BCI	brain–computer interface
CMD	cognitive motor dissociation
CRS-R	Coma Recovery Scale–Revised
DMN	default mode network
DoC	disorders of consciousness
ECG	electrocardiography
EEG	electroencephalography
ERP	event-related potential
FDG-PET	fluorodeoxyglucose positron emission tomography
fMRI	functional magnetic resonance imaging
fNIRS	functional near-infrared spectroscopy
HRV	heart rate variability
ICU	intensive care unit
MCS	minimally conscious state
MIBH	metabolic index of the best-preserved hemisphere
ML	machine learning
MRI	magnetic resonance imaging
ND	neurodegenerative disease
PCI	perturbational complexity index
PD	Parkinson’s disease
PET	positron emission tomography
PPG	photoplethysmography
rsfMRI	resting-state functional magnetic resonance imaging
SHAP	Shapley additive explanations
SVM	support vector machine
taVNS	transcutaneous auricular vagus nerve stimulation
TMS-EEG	transcranial magnetic stimulation combined with electroencephalography
UWS	unresponsive wakefulness syndrome
VS	vegetative state

## Appendix A

**Table A1:** PubMed literature search strategy.

Search Block (S), Thematic Domain, and Records Retrieved (*n*)	Exact PubMed Query
S1. Disorders of consciousness, awareness, and covert cognition; *n* = 2466	((“disorders of consciousness”[ti] OR “disorder of consciousness”[ti] OR “prolonged disorders of consciousness”[ti] OR “chronic disorders of consciousness”[ti]) OR (“minimally conscious state”[ti] OR “unresponsive wakefulness”[ti] OR “unresponsive wakefulness syndrome”[ti]) OR (“impaired consciousness”[ti] OR “disturbances of consciousness”[ti] OR (fluctuations[ti] AND consciousness[ti])) OR ((residual[ti] OR covert[ti]) AND (consciousness[ti] OR awareness[ti] OR cognition[ti])) OR (cognitive[ti] AND motor[ti] AND dissociation[ti]) OR (command[ti] AND following[ti]) OR “external responsiveness”[ti] OR (goal[ti] AND directed[ti] AND thinking[ti]) OR (detecting[ti] AND (awareness[ti] OR consciousness[ti])) OR (index[ti] AND consciousness[ti]) OR ((vegetative[ti] AND state[ti]) AND (awareness[ti] OR consciousness[ti] OR “minimally conscious”[ti] OR neurobehavioral[ti] OR “brain activity”[ti] OR detection[ti] OR “diffusion weighted imaging”[ti] OR “diffusion-weighted imaging”[ti])) OR (unconsciousness[ti] AND brain[ti])) AND 2000/01/01:2026/05/20[dp] AND english[la]
S2. Neurodegenerative disease, dementia, ALS, and impaired communication; *n* = 1778	((awareness[ti] AND (Alzheimer[ti] OR dementia[ti])) OR (“awareness of disease”[ti] AND dementia[ti]) OR (“severe Alzheimer”[ti] AND awareness[ti]) OR ((“fluctuating cognition”[ti] OR fluctuation[ti] OR “disturbances of consciousness”[ti] OR perfusion[ti] OR hallucinations[ti]) AND (dementia[ti] OR Alzheimer[ti] OR “Lewy bodies”[ti])) OR (P300[ti] AND (Alzheimer[ti] OR dementia[ti] OR “mild cognitive impairment”[ti])) OR ((“brain-computer interface”[ti] OR “brain computer interface”[ti]) AND (“amyotrophic lateral sclerosis”[ti] OR ALS[ti] OR paralysed[ti])) OR (“heart rate variability”[ti] AND (neurodegenerative[ti] OR cognition[ti] OR dementia[ti] OR “mild cognitive impairment”[ti])) OR (baroreflex[ti] AND dementia[ti]) OR (autonomic[ti] AND “mild cognitive impairment”[ti]) OR (neurocirculatory[ti] AND Parkinson[ti]) OR (“orthostatic hypotension”[ti] AND Parkinson[ti]) OR (“deep learning”[ti] AND (Alzheimer[ti] OR dementia[ti] OR “mild cognitive impairment”[ti])) OR (“multimodal deep learning”[ti] AND “cognitive decline”[ti]) OR (electroencephalography[ti] AND (Alzheimer[ti] OR dementia[ti]))) AND 2000/01/01:2026/05/20[dp] AND english[la]
S3. EEG, MRI/fMRI, fNIRS, PET, and brain networks; *n* = 836	(((EEG[ti] OR “EEG-derived”[ti] OR “EEG-based”[ti] OR “EEG signals”[ti] OR “EEG topographic maps”[ti] OR electroencephalography[ti] OR ERP[ti] OR “event-related potential”[ti] OR “event related potential”[ti] OR “event-related potentials”[ti] OR “event related potentials”[ti] OR P300[ti]) AND (consciousness[ti] OR “disorders of consciousness”[ti] OR “impaired consciousness”[ti] OR “covert cognition”[ti] OR “residual consciousness”[ti] OR “traumatic brain injury”[ti])) OR ((“single-trial decoding”[ti] OR “auditory novelty”[ti] OR “auditory regularities”[ti] OR “neural signatures”[ti] OR “spectral signatures”[ti] OR “brain complexity”[ti]) AND (consciousness[ti] OR “residual consciousness”[ti] OR “impaired consciousness”[ti] OR “brain networks”[ti] OR unresponsive[ti])) OR ((“functional connectivity”[ti] OR “intrinsic functional connectivity”[ti] OR “effective connectivity”[ti] OR “dynamic connectivity”[ti] OR “default mode network”[ti] OR “default-mode network”[ti] OR “default network”[ti] OR “brain functional networks”[ti] OR “brain connectivity”[ti] OR “brain networks”[ti] OR “default-mode network integrity”[ti]) AND (consciousness[ti] OR “disorders of consciousness”[ti] OR unresponsive[ti] OR recovery[ti] OR prognostic[ti])) OR ((“diffusion weighted imaging”[ti] OR “diffusion-weighted imaging”[ti] OR MRI[ti] OR fMRI[ti] OR rsfMRI[ti] OR “resting-state FMRI”[ti] OR “functional magnetic resonance”[ti] OR (FDG[ti] AND PET[ti]) OR “FDG PET”[ti] OR PET[ti] OR “functional neuroimaging”[ti]) AND (consciousness[ti] OR “disorders of consciousness”[ti] OR “vegetative state”[ti] OR “minimally conscious”[ti] OR “laboratory parameters”[ti])) OR ((fNIRS[ti] OR FNIRS[ti] OR “functional near-infrared spectroscopy”[ti]) AND (consciousness[ti] OR “cognitive motor dissociation”[ti] OR “severe brain injury”[ti] OR “residual cognition”[ti]))) AND 2000/01/01:2026/05/20[dp] AND english[la]
S4. Brain–computer interfaces, neuroprosthetics, and assistive communication; *n* = 901	(((“brain-computer interface”[ti] OR “brain computer interface”[ti] OR “brain-computer interfaces”[ti] OR “brain computer interfaces”[ti] OR BCI[ti]) AND (communication[ti] OR control[ti] OR “amyotrophic lateral sclerosis”[ti] OR ALS[ti] OR “disorders of consciousness”[ti] OR awareness[ti] OR prognosis[ti] OR tactile[ti] OR attention[ti])) OR “brain-computer interface technology”[ti] OR (“hybrid BCI”[ti] AND (consciousness[ti] OR awareness[ti] OR “number processing”[ti] OR “mental calculation”[ti])) OR (hybrid[ti] AND BCI[ti]) OR (“P300-based”[ti] AND (“brain-computer interface”[ti] OR “brain computer interface”[ti] OR BCI[ti])) OR ((implanted[ti] OR implantable[ti]) AND (“brain-computer interface”[ti] OR “brain computer interface”[ti]) AND (ALS[ti] OR “locked-in”[ti])) OR ((neuroprosthesis[ti] OR neuroprosthetic[ti] OR neuroprosthetics[ti] OR “motor neuroprosthesis”[ti]) AND (paralysis[ti] OR paralysed[ti] OR paralyzed[ti]))) AND 2000/01/01:2026/05/20[dp] AND english[la]
S5. Artificial intelligence, machine learning, and multimodal prognostic models; *n* = 2417	(((“artificial intelligence”[ti] OR “machine learning”[ti] OR “deep learning”[ti]) AND (consciousness[ti] OR “disorders of consciousness”[ti] OR “prolonged disorders of consciousness”[ti] OR EEG[ti] OR Alzheimer[ti] OR dementia[ti] OR “mild cognitive impairment”[ti])) OR ((multimodal[ti] OR multicentre[ti] OR explainable[ti]) AND (“machine learning”[ti] OR “deep learning”[ti] OR markers[ti] OR prognostic[ti] OR prediction[ti] OR model[ti]) AND (consciousness[ti] OR “cognitive decline”[ti])) OR ((multi[ti] AND domain[ti]) AND prognostic[ti] AND consciousness[ti]) OR ((neuroprognostic[ti] OR personalized[ti] OR digital[ti]) AND (model[ti] OR models[ti] OR tracking[ti] OR twin[ti]) AND consciousness[ti]) OR ((data[ti] AND mining[ti]) AND “vegetative state”[ti]) OR (diagnosis[ti] AND framework[ti] AND (EEG[ti] OR consciousness[ti])) OR (dynamic[ti] AND complex[ti] AND patterns[ti] AND (EEG[ti] OR consciousness[ti])) OR (prediction[ti] AND “cognitive decline”[ti] AND Alzheimer[ti])) AND 2000/01/01:2026/05/20[dp] AND english[la]
S6. Autonomic biomarkers, HRV, baroreflex, and vagus nerve stimulation; *n* = 572	((“heart rate variability”[ti] AND (consciousness[ti] OR “disorders of consciousness”[ti] OR cognition[ti] OR behavior[ti] OR neurodegenerative[ti] OR dementia[ti] OR “mild cognitive impairment”[ti] OR “Lewy bodies”[ti] OR “brain processing”[ti] OR “affective videos”[ti])) OR (“central autonomic network”[ti] AND (consciousness[ti] OR prognosis[ti])) OR (brain[ti] AND heart[ti] AND interplay[ti]) OR (baroreflex[ti] AND (dementia[ti] OR Alzheimer[ti])) OR (autonomic[ti] AND (“mild cognitive impairment”[ti] OR consciousness[ti] OR “disorders of consciousness”[ti])) OR ((neurocirculatory[ti] OR “orthostatic hypotension”[ti]) AND Parkinson[ti]) OR (“auditory stimulation”[ti] AND (autonomic[ti] OR consciousness[ti] OR “disorders of consciousness”[ti])) OR ((vagus[ti] OR vagal[ti] OR taVNS[ti] OR transauricular[ti] OR transcutaneous[ti]) AND (stimulation[ti] OR modulation[ti] OR nerve[ti]) AND (consciousness[ti] OR “disorders of consciousness”[ti] OR “prolonged disorders of consciousness”[ti] OR recovery[ti])) OR (magnetic[ti] AND modulation[ti] AND vagus[ti] AND consciousness[ti])) AND 2000/01/01:2026/05/20[dp] AND english[la]
Final combined search; *n* = 7717	S1 OR S2 OR S3 OR S4 OR S5 OR S6
Selected articles; *n* = 111	(References PMID list) NOT (Final combined search) → *n* = 0;

Note: PubMed title-field search strategy for identifying records on disorders of consciousness and related constructs. The query combined terms referring to disorders of consciousness, minimally conscious state, unresponsive wakefulness syndrome, impaired or fluctuating consciousness, covert or residual awareness or cognition, cognitive–motor dissociation, command-following, external responsiveness, goal-directed thinking, detection of awareness or consciousness, consciousness indices, vegetative state, and brain-related unconsciousness. The search was limited to English-language records published between 1 January 2000 and 20 May 2026.

**Table A2:** Targeted critical appraisal of selected studies important to the diagnostic, prognostic, and translational arguments of the review.

Study	Clinical Context and Method	Key Strength and Relevance to Review	Main Limitation for Translation
Foundational Acquired DoC/Covert Awareness/Command-Following			
Schnakers et al., 2009 [3]	Acquired DoC; standardized neurobehavioral assessment with CRS-R in 103 patients. Clinical consensus classified 44 patients as VS, 41 as MCS, and 18 as uncertain; CRS-R reclassified 18 of 44 VS patients as MCS.	Establishes the clinical problem of behavioral misclassification and supports the need for objective multimodal assessment when motor output and arousal fluctuate.	Behavioral reference standards remain influenced by fluctuation, repeated assessment, and examiner-dependent factors.
Owen et al., 2006 [11]	Vegetative state; active fMRI mental imagery paradigm in a single patient fulfilling VS criteria, with comparison to healthy control activation patterns.	Landmark proof of principle that covert awareness can be detected even when purposeful behavior is absent.	Single-patient demonstration; requires task comprehension, preserved imagery capacity, and advanced neuroimaging infrastructure.
Monti et al., 2010 [12]	DoC; active fMRI mental imagery and communication paradigm in 54 patients with VS or MCS. Five patients showed willful modulation of brain activity.	Provides cohort-level evidence that command-following and, in selected cases, communication may be detectable without behavioral output.	Responders were a minority; scanner paradigms may be difficult to apply in frail, medically unstable, or fluctuating patients.
Cruse et al., 2011 [13], with Goldfine et al., 2013 reanalysis [14] and Cruse et al., 2013 author reply [15]	Vegetative state; bedside EEG motor imagery paradigm in 16 VS patients and 12 controls, followed by reanalysis and author reply concerning statistical inference, permutation testing, independence assumptions, and validation of EEG command-following.	Important example of bedside EEG detection of covert command-following and of the methodological debate surrounding machine-assisted interpretation of EEG responses in DoC. This paired case supports the review’s argument that positive machine-assisted EEG findings require transparent preprocessing, artifact control, appropriate statistical testing, and independent validation.	Interpretation is sensitive to task structure, signal quality, preprocessing choices, statistical assumptions, and classifier strategy; the subsequent reanalysis and author reply show that replication and convergent evidence are essential before clinical translation.
EEG, MRI/fMRI, fNIRS, PET, and brain networks			
Casali et al., 2013 [26]	Conscious, unconscious, anesthetized, and brain-injured states; TMS-EEG perturbational complexity index (PCI), with 208 TMS-EEG sessions in 52 subjects.	Introduced PCI as a physiological index of consciousness not dependent on behavior, with strong conceptual relevance for patients unable to communicate.	Requires specialized stimulation, EEG acquisition, and analysis; direct validation in progressive neurodegenerative cohorts remains necessary.
Casarotto et al., 2016 [27]	Unresponsive patients; PCI validation using a benchmark sample of 150 healthy controls and communicative brain-injured subjects, followed by application to 81 non-communicative DoC patients, including 38 MCS and 43 VS patients.	Provides an important validation step for stratifying unresponsive patients with a physiological measure less dependent on overt behavior.	Strongest evidence concerns acquired DoC; transfer to neurodegenerative disease requires disease-focused validation.
Vanhaudenhuyse et al., 2010 [28]	Non-communicative brain-damaged patients; resting-state fMRI default mode network connectivity in 14 patients and 14 healthy controls.	Shows that default network connectivity reflects level of consciousness and supports large-scale network integrity as a clinically relevant marker.	Small and heterogeneous cohort; network measures are best interpreted as part of multimodal assessment and not as stand-alone diagnostic markers.
Hermann et al., 2021 [76]	DoC; multimodal FDG-PET and EEG assessment in 52 patients, including 21 VS and 31 MCS patients. FDG-PET MIBH showed AUC 0.821, sensitivity 79%, and specificity 78%; adding EEG increased sensitivity for MCS detection.	Strong example of complementary multimodal diagnosis and prognosis, combining metabolic imaging with electrophysiology. It supports the review’s argument that markers from a single modality may be strengthened by multimodal integration.	FDG-PET availability, cost, and center-dependent workflows may limit routine or repeated bedside use; transfer to neurodegenerative disease requires separate validation.
Kazazian et al., 2024 [36]	Acute severe brain injury; fNIRS assessment across healthy participant substudies and 3 acutely brain-injured ICU patients.	Supports fNIRS as a portable hemodynamic tool for detecting sensory, resting-state, and command-driven activity at the bedside.	Patient evidence remains early; larger ICU and neurodegenerative validation cohorts are needed.
BCI, ALS, locked-in states, and neuroprosthetic communication			
Sellers and Donchin, 2006 [17]	ALS; P300 BCI four-choice communication interface tested in 3 ALS patients and 3 non-ALS controls.	Early direct evidence that BCI can support communication in patients with preserved cognition but impaired motor output.	Small proof-of-principle sample; performance in advanced locked-in states may differ because of fatigue, attention, and disease progression.
Wolpaw et al., 2018 [41]	Advanced ALS; EEG-based home BCI use. Forty-two patients consented, 39 met criteria, 37 were assessed, 27 had the system placed at home, and 14 completed training and used it independently.	Strong translational evidence because it addresses independent home use, usability, caregiver support, and long-term communication outside the laboratory.	Feasibility depends on disease stage, technical support, caregiver involvement, and user burden.
Vansteensel et al., 2016 [43]	Late-stage ALS/locked-in state; fully implanted BCI for home communication in a single patient. Independent typing was achieved 28 weeks after electrode placement, at approximately two letters per minute.	Landmark demonstration of implanted BCI communication in a clinically relevant locked-in state.	Highly selected single-patient invasive study; scalability and accessibility remain limited.
Vansteensel et al., 2024 [42]	Advanced ALS; 7-year follow-up of independent at-home implanted BCI use in the same clinical context.	Particularly relevant for neurodegenerative disease because it addresses durability of BCI use during progressive motor decline.	Long-term reliability may change with disease progression and neural signal changes; individualized monitoring remains necessary.
AI, machine learning, and multimodal prognostic models			
Toppi et al., 2024 [20]	DoC; routine resting EEG in 58 UWS/MCS patients with 3-month follow-up; EEG spectral and network features were used in machine-learning models.	Clinically relevant because EEG is accessible and suitable for repeated assessment; reported performance reached up to 85% accuracy for outcome prediction and 76% for UWS/MCS recognition.	Requires external validation and careful handling of etiology-dependent EEG patterns before broader clinical use.
Yang et al., 2024 [21]	DoC; rs-fMRI deep learning framework in 140 participants, including 76 UWS, 25 MCS, and 39 controls from three sites; independent CMD dataset of 11 DoC patients.	Central AI study. DeepDOC achieved AUC 0.927 and accuracy 0.861 for distinguishing MCS from UWS, and AUC 1.0 with accuracy 0.909 for CMD identification.	Imaging model; requires interpretability, prospective testing, and validation across etiologies, scanners, and clinical settings.
Mou et al., 2026 [50]	DoC; explainable multimodal machine learning in a multicenter cohort of 583 patients, including 247 UWS/VS and 336 MCS patients. Clinical-only modelling used the full cohort, whereas EEG and combined clinical-EEG models were developed in the EEG subset of 158 patients; external validation included 70 patients.	Strong translational anchor because it combines multicenter clinical data, EEG features, SHAP interpretability, and external validation.	Model performance was clinically realistic but not definitive; transfer to neurodegenerative disease requires separate validation.
Manasova et al., 2026 [51]	DoC; multicenter multimodal neuroimaging and neurophysiology including EEG, anatomical MRI, rs-fMRI, diffusion MRI, and FDG-PET.	Supports the review’s multimodal argument by showing that diagnostic and prognostic information differs across modalities and can be strengthened by integration.	Not all patients had all modalities or prognostic information; multimodal workflows may be difficult to implement uniformly in routine practice.
Wu H. et al., 2025 [53]	Chronic DoC; retrospective multimodal prognostic model in 43 patients, including 19 with favorable prognosis and 24 with poor prognosis. The model integrated CRS-R, pituitary-related hormones, and rs-fMRI features.	Useful example of multimodal prognosis beyond neuroimaging alone; reported accuracy was 0.91, with sensitivity 0.84 and specificity 0.96.	Relatively small retrospective cohort; larger external validation is needed before clinical transfer.
Autonomic biomarkers			
Riganello et al., 2018 [69]	DoC; HRV entropy/complexity index in 14 UWS and 16 MCS patients. fMRI connectivity was analyzed in a subgroup of 10 UWS and 11 MCS patients with sufficient image quality.	Strong autonomic anchor because it links HRV complexity with clinical consciousness level and central autonomic network connectivity. The One-R classifier selected the long-timescale complexity index as the best discriminator, with 90% accuracy after 10-fold cross-validation.	HRV is best interpreted as an adjunctive marker because it is influenced by sedation, medication, systemic physiology, circadian effects, and autonomic comorbidity.
Liu et al., 2022 [74]	Neurodegenerative diseases; systematic review and meta-analysis of HRV in relation to cognition and behavior. The review included 27 studies, with cross-sectional quantitative assessment across 18 studies.	Provides direct neurodegenerative context for autonomic biomarkers. A moderate association was reported between higher HRV and better cognitive or behavioral scores, with r = 0.25.	Heterogeneity across diseases, HRV protocols, medication exposure, and cognitive or behavioral outcomes limit specific interpretation.

Note: This table provides a targeted appraisal of selected anchor studies rather than a formal risk-of-bias assessment of all cited literature. The limitations are interpreted in relation to translation from acquired disorders of consciousness to neurodegenerative disease. DoC, disorders of consciousness; VS, vegetative state; UWS, unresponsive wakefulness syndrome; MCS, minimally conscious state; CMD, cognitive motor dissociation; CRS-R, Coma Recovery Scale–Revised; EEG, electroencephalography; TMS-EEG, transcranial magnetic stimulation combined with electroencephalography; MRI, magnetic resonance imaging; fMRI, functional magnetic resonance imaging; rs-fMRI, resting-state functional magnetic resonance imaging; fNIRS, functional near-infrared spectroscopy; PET, positron emission tomography; FDG-PET, fluorodeoxyglucose positron emission tomography; MIBH, metabolic index of the best hemisphere; PCI, perturbational complexity index; HRV, heart rate variability; BCI, brain–computer interface; ALS, amyotrophic lateral sclerosis; ICU, intensive care unit; AI, artificial intelligence; SHAP, Shapley additive explanations; One-R, one-rule classifier; AUC, area under the curve.

**Table A3:** QUADAS-2-based assessment of selected studies in disorders of consciousness.

Study	Method Assessed/Comparator	Patient Selection	Index Test	Reference Standard	Flow and Timing
Schnakers et al., 2009 [3]	Clinical consensus diagnosis compared with CRS-R-based standardized neurobehavioral assessment	PS-Q1 Unclear; PS-Q2 Yes; PS-Q3 Yes; RoB Unclear; App Low	IT-Q1 Yes; IT-Q2 Unclear; RoB Low/Unclear; App Low	RS-Q1 Yes; RS-Q2 No/Unclear; RoB High; App Low	FT-Q1 Yes; FT-Q2 Yes; FT-Q3 Yes; FT-Q4 Yes; RoB Low
Rationale	The study prospectively assessed 103 patients with acquired DoC and directly compared routine clinical consensus diagnosis with CRS-R-derived diagnosis. Case–control design was avoided and exclusions were clinically appropriate, but consecutive or random enrolment was not explicitly stated. The clinical consensus diagnosis was recorded before CRS-R assessment, supporting independence of the index test. However, CRS-R raters were explicitly not masked to the clinical consensus diagnosis, increasing risk of bias in the reference-standard domain. Timing was appropriate because clinical consensus included observations from the previous 24 h and was provided just before CRS-R assessment. The study remains highly relevant because it directly addresses diagnostic misclassification between VS/UWS and MCS, but single CRS-R assessment and fluctuation of arousal remain interpretive limitations.				
Monti et al., 2010 [12]	Active fMRI mental-imagery paradigm for willful modulation of brain activity, with bedside clinical diagnosis and follow-up behavioral assessment as comparator	PS-Q1 No; PS-Q2 Yes; PS-Q3 Unclear; RoB High; App Low	IT-Q1 Unclear; IT-Q2 Yes; RoB Unclear; App Low	RS-Q1 Unclear; RS-Q2 Unclear; RoB Unclear; App Unclear	FT-Q1 Unclear; FT-Q2 Yes; FT-Q3 Unclear; FT-Q4 Yes; RoB Unclear
Rationale	The study used a convenience sample of 54 patients with disorders of consciousness, including patients clinically diagnosed as VS or MCS. Case–control design was avoided, but enrolment was not consecutive or random, and MRI feasibility may have selected for patients able to undergo scanning. The index test was based on established motor and spatial imagery paradigms, predefined regions of interest, and a prespecified statistical threshold, supporting methodological relevance of the fMRI procedure. However, masking of fMRI interpretation to clinical diagnosis is not clearly stated. Bedside clinical assessment is an appropriate clinical comparator but an imperfect reference standard for covert awareness, because the target condition includes willful brain modulation that may be present without behavioral output. Flow was broadly complete for the 54-patient fMRI cohort, but timing and uniformity of behavioral reassessment were not fully clear, especially because additional bedside testing in some cases followed fMRI findings.				
Cruse et al., 2011 [13]	Bedside EEG motor-imagery paradigm for covert command-following, compared with repeated CRS-R-based behavioral assessment defining vegetative state	PS-Q1 No; PS-Q2 Yes; PS-Q3 Unclear; RoB High; App Low	IT-Q1 Unclear; IT-Q2 Yes; RoB Low/Unclear; App Low	RS-Q1 Unclear; RS-Q2 Unclear; RoB Unclear; App Unclear	FT-Q1 Yes; FT-Q2 Yes; FT-Q3 Yes; FT-Q4 Yes; RoB Low
Rationale	The study assessed 16 patients who met CRS-R criteria for vegetative state and 12 healthy controls. Patient selection was based on a convenience sample, which increases selection bias, although the patient cohort was clinically relevant to the review question and did not use a classical case–control design. The EEG index test was methodologically well specified, using predefined motor-imagery commands, motor-area electrodes, μ/β band features, blockwise cross-validation, binomial significance testing, and baseline comparison. However, blinding of EEG interpretation to clinical status was not clearly reported. CRS-R was appropriate for behavioral classification of vegetative state, but it is an imperfect reference standard for covert awareness because the target condition is command-following without overt behavior. Flow and timing were acceptable because all patients underwent repeated CRS-R assessment during the 4–5-day admission and were included in the EEG analysis.				
Goldfine et al., 2013 [14]	Reanalysis of the Cruse et al. EEG motor-imagery classifier using alternative cross-validation, permutation testing, FDR correction, and univariate task–rest analysis	PS-Q1 No/Unclear; PS-Q2 Yes; PS-Q3 Unclear; RoB Unclear; App Low	IT-Q1 Unclear; IT-Q2 Yes/Unclear; RoB Unclear; App Low	RS-Q1 Unclear; RS-Q2 Unclear; RoB Unclear; App Unclear	FT-Q1 Unclear; FT-Q2 Yes; FT-Q3 Yes; FT-Q4 Yes; RoB Low/Unclear
Rationale	This article is a methodological reanalysis rather than an independent diagnostic accuracy study. Patient selection was inherited from the original Cruse et al. cohort, so selection bias cannot be independently resolved. The reanalysis directly addresses the statistical robustness of the EEG motor-imagery index test by accounting for correlations between trials and blocks, using alternative block-pair cross-validation, permutation testing, FDR correction, and univariate physiological analyses. These methods raised concerns that the original positive patient classifications may have depended on statistical assumptions not met by the patient EEG data. However, the study did not introduce a new independent clinical reference standard for covert awareness, and its role is best interpreted as a robustness and validity critique of the original classifier rather than as a standalone diagnostic study.				
Casali et al., 2013 [26]	TMS-EEG perturbational complexity index (PCI) compared with controlled conscious/unconscious states and CRS-R-based clinical categories in brain-injured patients	PS-Q1 No; PS-Q2 No/Unclear; PS-Q3 Yes; RoB High/Unclear; App Low	IT-Q1 Unclear; IT-Q2 Yes; RoB Low/Unclear; App Low	RS-Q1 Yes; RS-Q2 Unclear; RoB Low/Unclear; App Low	FT-Q1 Yes; FT-Q2 Yes; FT-Q3 Yes/Unclear; FT-Q4 Yes/Unclear; RoB Low/Unclear
Rationale	This hypothesis-generating validation study introduced PCI as a TMS-EEG measure of cortical complexity intended to assess consciousness independently of sensory processing and behavior. Patient selection was not consecutive or random and the design was partly case–control-like, because PCI was tested across predefined conscious, unconscious, and clinically diagnosed groups. However, exclusions and protocol constraints were appropriate for TMS-EEG. The index test was technically well specified, including TMS perturbation, EEG source modelling, nonparametric statistics, Lempel–Ziv complexity, and normalization procedures. Reference standards were appropriate for the studied conditions, including controlled wakefulness, sleep, anesthesia/MOAAS, locked-in syndrome, and repeated CRS-R-based clinical diagnosis in patients. Main limitations are the open-label design, small brain-injured subgroup, mixed prior and newly acquired datasets, and the need for independent validation in patients with dissociation between behavior and consciousness.				
Casarotto et al., 2016 [27]	TMS-EEG perturbational complexity index using PCImax and an independently validated cutoff, compared with subjective-report benchmark conditions and repeated CRS-R-based clinical diagnosis in DoC patients	PS-Q1 Unclear; PS-Q2 Yes/Unclear; PS-Q3 Yes; RoB Unclear; App Low	IT-Q1 Yes/Unclear; IT-Q2 Yes; RoB Low/Unclear; App Low	RS-Q1 Yes/Unclear; RS-Q2 Yes/Unclear; RoB Low/Unclear; App Low	FT-Q1 Yes; FT-Q2 Yes; FT-Q3 Yes; FT-Q4 Yes; RoB Low
Rationale	The study strengthened PCI validation by first deriving an empirical cutoff in a benchmark population of healthy controls and communicative brain-injured subjects who could provide immediate or delayed subjective reports, and then applying this cutoff to 81 noncommunicative DoC patients. Patient selection was not explicitly consecutive or random and the benchmark design was enriched for known conscious and unconscious conditions, but exclusions were appropriate for TMS-EEG safety and interpretability. The index test was well specified, with MRI-guided TMS, EEG preprocessing, PCImax calculation, and ROC-derived cutoff applied to the DoC cohort. Reference standards were clinically meaningful, including the subjective report in the benchmark population and repeated CRS-R over 1 week in DoC patients, although behavioral diagnosis remains imperfect for covert consciousness in VS/UWS. Flow and timing were strong because TMS-EEG was scheduled during the same evaluation week, sedation was withdrawn before testing, vigilance was monitored, and all DoC patients received repeated CRS-R assessment.				
Vanhaudenhuyse et al., 2010 [28]	Resting-state fMRI default mode network connectivity compared with CRS-R-based clinical level of consciousness in noncommunicative brain-damaged patients	PS-Q1 Unclear; PS-Q2 Yes/Unclear; PS-Q3 Unclear; RoB Unclear; App Low	IT-Q1 Unclear; IT-Q2 Yes/Unclear; RoB Low/Unclear; App Low	RS-Q1 Yes/Unclear; RS-Q2 Yes/Unclear; RoB Low/Unclear; App Low	FT-Q1 Yes; FT-Q2 Yes; FT-Q3 Yes; FT-Q4 Yes/Unclear; RoB Low/Unclear
Rationale	The study examined resting-state DMN connectivity in 14 brain-damaged patients and 14 healthy controls, showing that DMN connectivity decreased in proportion to clinical impairment of consciousness and that precuneus connectivity differentiated MCS from unconscious patients. Patient selection was not clearly consecutive or random and the sample was small, but the population was clinically relevant to DoC assessment. The index test was technically well specified, using resting-state fMRI, probabilistic ICA, and automated template matching based on an independent DMN template; however, no validated diagnostic cutoff was provided and blinding to clinical diagnosis was not clearly stated. The reference standard was appropriate for behavioral diagnosis because patients were repeatedly assessed with CRS-R and the Glasgow Liège Scale on the day of scanning and in the surrounding weeks, although behavioral assessment remains imperfect for covert consciousness. Flow and timing were acceptable, but the study should be interpreted mainly as group-level evidence supporting DMN connectivity as a potential paraclinical marker rather than as a validated patient-level diagnostic test.				
Hermann et al., 2021 [76]	FDG-PET metabolic index of the best preserved hemisphere, EEG-based classification, and combined PET/EEG multimodal assessment compared with repeated CRS-R-based diagnosis and 6-month recovery of command-following	PS-Q1 Yes/Unclear; PS-Q2 Yes; PS-Q3 Yes/Unclear; RoB Low/Unclear; App Low	IT-Q1 Yes/Unclear; IT-Q2 Yes; RoB Low/Unclear; App Low	RS-Q1 Yes; RS-Q2 Yes/Unclear; RoB Low/Unclear; App Low	FT-Q1 Yes; FT-Q2 Yes; FT-Q3 Yes; FT-Q4 Yes/Unclear; RoB Low/Unclear
Rationale	This prospective single-center study included prolonged DoC patients admitted for specialized diagnosis and compared FDG-PET MIBH, EEG-based classification, and their combination with the best state of consciousness observed across repeated CRS-R assessments. Patient selection was clinically relevant and not case–control, although only patients eligible for PET, including nonventilated patients, could be scanned and some recordings were excluded for quality reasons. The index tests were well specified: PET-MIBH used a previously published out-of-sample threshold, and EEG classification used a predefined feature/SVM pipeline trained externally. CRS-R was an appropriate behavioral reference standard for MCS vs VS/UWS, although repeated assessment was limited in some VS/UWS patients and cannot fully exclude covert cognition. Flow and timing were generally strong, with PET and EEG performed during the diagnostic admission and short delays from best CRS-R, but not always on the same day, and the final EEG/PET analyses included slightly different subsets.				
Toppi et al., 2024 [20]	Routine resting-state EEG spectral and connectivity features with SVM models for 3-month outcome prediction and UWS/MCS discrimination, compared with CRS-R-based diagnosis and 3-month CRS-R-based outcome	PS-Q1 Yes; PS-Q2 Yes; PS-Q3 Yes/Unclear; RoB Low/Unclear; App Low	IT-Q1 No/Unclear; IT-Q2 No/Unclear; RoB High/Unclear; App Low	RS-Q1 Yes; RS-Q2 Unclear; RoB Low/Unclear; App Low	FT-Q1 Yes; FT-Q2 Yes; FT-Q3 Yes; FT-Q4 Unclear; RoB Low/Unclear
Rationale	The study consecutively enrolled 58 patients with DoC diagnosed as UWS or MCS and followed them for 3 months, making patient selection more robust than in many exploratory DoC studies. The cohort was clinically relevant and did not use a case–control design. The EEG index test was based on routine 19-channel resting-state EEG, spectral features, connectivity measures, graph indices, and SVM classification, supporting bedside feasibility and applicability. However, the feature selection and classification procedures were exploratory, without external validation, and may overestimate model performance; therefore, the index-test domain carries higher/unclear risk of bias. CRS-R was an appropriate behavioral reference standard for UWS/MCS diagnosis and EMCS/outcome classification, although blinding to EEG was not clearly reported and CRS-R remains imperfect for covert consciousness. Flow and timing were appropriate because CRS-R was administered before EEG and follow-up was performed at 3 months, but not all patients contributed to every EEG feature analysis because some recordings were excluded from spectral or connectivity analyses.				
Kazazian et al., 2024 [36]	Bedside fNIRS battery assessing resting-state networks, sensory/auditory processing, and active motor-imagery command-following, compared with bedside behavioral diagnosis/CRS-R in acute severe brain injury	PS-Q1 Unclear; PS-Q2 Yes/Unclear; PS-Q3 Unclear; RoB Unclear/High; App Low	IT-Q1 Unclear; IT-Q2 Yes/Unclear; RoB Unclear; App Low	RS-Q1 Yes/Unclear; RS-Q2 Unclear; RoB Unclear; App Low/Unclear	FT-Q1 Yes/Unclear; FT-Q2 Yes; FT-Q3 Yes/Unclear; FT-Q4 Yes/Unclear; RoB Unclear
Rationale	This proof-of-principle study evaluated whether fNIRS can detect neural signatures of conscious processing in healthy participants and then applied the approach to 3 acutely unresponsive ICU patients. Patient selection was not clearly consecutive or random, and the clinical sample was very small, limiting diagnostic generalizability. The index test was clinically relevant and technically well described, using portable fNIRS, predefined task paradigms, short-channel regression, GLM analysis, and conservative activation criteria requiring concurrent HbO and HbR changes. However, patient-level diagnostic thresholds and blinding to behavioral diagnosis were not clearly established. CRS-R/bedside behavioral assessment was an appropriate clinical comparator, but it is intrinsically limited for covert awareness and cognitive-motor dissociation. Flow was acceptable for the case-series aim, but not all paradigms yielded or included complete data across all patients, and the study should be interpreted as feasibility evidence rather than a validated diagnostic accuracy study.				
Manasova et al., 2026 [51]	Multimodal EEG, FDG-PET, fMRI, dMRI and aMRI markers integrated with interpretable Random Forest machine learning for diagnostic classification and prognosis, compared with CRS-R-based diagnosis and clinical outcome	PS-Q1 Unclear; PS-Q2 Yes; PS-Q3 Yes/Unclear; RoB Unclear; App Low	IT-Q1 Unclear/NA; IT-Q2 Unclear; RoB Unclear; App Low	RS-Q1 Yes/Unclear; RS-Q2 Unclear; RoB Low/Unclear; App Low	FT-Q1 Yes/Unclear; FT-Q2 Yes; FT-Q3 Unclear; FT-Q4 Unclear; RoB Unclear
Rationale	This large multicenter study integrated several electrophysiological and neuroimaging modalities under a common interpretable machine-learning framework to classify UWS versus MCS and to predict patient evolution. The population was clinically relevant and case–control design was avoided, but consecutive or random enrolment was not clearly stated and modality availability was heterogeneous. The index tests were technically well described and included EEG-RS, EEG-LG, fMRI-RS, FDG-PET, dMRI and aMRI, but the final diagnostic and prognostic outputs were data-driven machine-learning models rather than prespecified single-modality clinical thresholds. CRS-R-based best clinical diagnosis was an appropriate reference standard for UWS/MCS classification, and clinical follow-up was appropriate for prognosis, but behavioral labels remain imperfect for covert consciousness and long-term outcome assessment relied on limited follow-up procedures. Flow was acceptable for a sparse multimodal dataset, yet not all patients had all modalities or prognostic information, making this best interpreted as a robust translational multimodal modelling study rather than a fully validated clinical diagnostic test.				
Yang et al., 2024 [21]	DeepDOC rs-fMRI deep-learning model for MCS/UWS classification and CMD identification, compared with CRS-R-based diagnosis and BCI-based CMD classification	PS-Q1 Unclear; PS-Q2 Yes/Unclear; PS-Q3 No/Unclear; RoB High/Unclear; App Low/Unclear	IT-Q1 Yes/Unclear; IT-Q2 Unclear; RoB Unclear/High; App Low	RS-Q1 Yes/Unclear; RS-Q2 Yes/Unclear; RoB Low/Unclear; App Low	FT-Q1 Unclear; FT-Q2 Yes; FT-Q3 Yes/Unclear; FT-Q4 No/Unclear; RoB Unclear/High
Rationale	This retrospective multicenter rs-fMRI study developed a cascade deep-learning model to distinguish MCS from UWS and to identify CMD-like cases in an independent BCI-labelled dataset. The study used data from three centers and reported strong performance for MCS/UWS classification and CMD identification. However, patient enrolment was not clearly consecutive or random, the MCS and CMD samples were small, and patients with extensive focal lesions or segmentation/normalization problems were excluded, limiting generalizability to the broader DoC population. The index test was technically well described and included an automated rs-fMRI deep-learning pipeline, but the MCS/UWS threshold was optimized within the modelling procedure rather than externally prespecified. CRS-R was an appropriate behavioral reference standard for MCS/UWS classification, and BCI provided a relevant comparator for CMD, although both remain imperfect for covert awareness. Flow and timing are less clear because the study used retrospective datasets collected over several years, and not all initially eligible participants were included in the final analysis.				
Mou et al., 2026 [50]	Explainable ML models based on clinical features, EEG features, and combined clinical-EEG features, compared with repeated CRS-R-based UWS/VS versus MCS diagnosis	PS-Q1 Unclear; PS-Q2 Yes; PS-Q3 Yes/Unclear; RoB Low/Unclear; App Low	IT-Q1 Yes/Unclear; IT-Q2 Yes; RoB Low/Unclear; App Low	RS-Q1 Yes; RS-Q2 Yes/Unclear; RoB Low/Unclear; App Low	FT-Q1 Yes; FT-Q2 Yes; FT-Q3 Yes; FT-Q4 Yes/Unclear; RoB Low/Unclear
Rationale	This large multicenter study developed explainable ML models for UWS/VS versus MCS classification using clinical variables, EEG features, and their combination. The full cohort included 583 patients, whereas the EEG and combined clinical-EEG models were developed in the EEG subset of 158 patients and externally validated in 70 patients. Patient selection was clinically relevant and case–control design was avoided, but consecutive or random recruitment was not clearly stated and exclusions related to missing data, epilepsy, or poor EEG quality may limit generalizability. The index test was well specified, using predefined EEG preprocessing, spectral and connectivity features, LASSO feature selection, multiple ML algorithms, a fixed 0.5 probability threshold, calibration analysis, decision curve analysis, external validation, and SHAP interpretability. CRS-R was an appropriate behavioral reference standard, strengthened by five assessments within 10 days and independent evaluation by two trained neurologists, although behavioral labels remain imperfect for covert consciousness. Flow and timing were appropriate because EEG and clinical/laboratory data were collected close to CRS-R assessment, but not all patients contributed to the EEG-based models.				

Note: QUADAS-2 signaling questions were abbreviated as follows. PS-Q1, consecutive or random sample enrolled; PS-Q2, case–control design avoided; PS-Q3, inappropriate exclusions avoided. IT-Q1, index test interpreted without knowledge of the reference standard; IT-Q2, threshold pre-specified. RS-Q1, reference standard likely to correctly classify the target condition; RS-Q2, reference standard interpreted without knowledge of the index test. FT-Q1, appropriate interval between index test and reference standard; FT-Q2, all patients received a reference standard; FT-Q3, all patients received the same reference standard; FT-Q4, all patients included in the analysis. RoB, risk of bias; App, applicability concern within the corresponding QUADAS-2 domain; NA, not applicable. Compound judgements such as Low/Unclear or High/Unclear indicate mixed evidence within a domain or partial applicability of the signaling question to the study design. Judgements were based on the published full texts and were adapted to the diagnostic, prognostic, methodological, or translational role of each study in this narrative review. Because several included studies were proof-of-principle, methodological reanalysis, multimodal modelling, or prognostic studies rather than conventional diagnostic accuracy studies, QUADAS-2 was used as a structured assessment tool rather than as a strict study-ranking instrument.

## References

[1] Laureys S. (2005). Death, Unconsciousness and the Brain. Nat. Rev. Neurosci..

[2] Giacino J.T., Ashwal S., Childs N., Cranford R., Jennett B., Katz D.I., Kelly J.P., Rosenberg J.H., Whyte J., Zafonte R.D. (2002). The Minimally Conscious State. Neurology.

[3] Schnakers C., Vanhaudenhuyse A., Giacino J., Ventura M., Boly M., Majerus S., Moonen G., Laureys S. (2009). Diagnostic Accuracy of the Vegetative and Minimally Conscious State: Clinical Consensus versus Standardized Neurobehavioral Assessment. BMC Neurol..

[4] Ballard C.G., Court J.A., Piggott M., Johnson M., O’Brien J., McKeith I., Holmes C., Lantos P., Jaros E., Perry R. (2002). Disturbances of Consciousness in Dementia with Lewy Bodies Associated with Alteration in Nicotinic Receptor Binding in the Temporal Cortex. Conscious. Cogn..

[5] Dourado M., Marinho V., Soares C., Engelhardt E., Laks J. (2007). Awareness of Disease in Alzheimer’s Dementia: Description of a Mild to Moderate Sample of Patient and Caregiver Dyads in Brazil. Int. Psychogeriatr..

[6] Wolpaw J.R., Birbaumer N., McFarland D.J., Pfurtscheller G., Vaughan T.M. (2002). Brain–Computer Interfaces for Communication and Control. Clin. Neurophysiol..

[7] Bradshaw J. (2004). Fluctuating Cognition in Dementia with Lewy Bodies and Alzheimer’s Disease Is Qualitatively Distinct. J. Neurol. Neurosurg. Psychiatry.

[8] Walker M.P., Ayre G.A., Cummings J.L., Wesnes K., McKeith I.G., O’Brien J.T., Ballard C.G. (2000). Quantifying Fluctuation in Dementia with Lewy Bodies, Alzheimer’s Disease, and Vascular Dementia. Neurology.

[9] O’Brien J.T., Firbank M.J., Mosimann U.P., Burn D.J., McKeith I.G. (2005). Change in Perfusion, Hallucinations and Fluctuations in Consciousness in Dementia with Lewy Bodies. Psychiatry Res. Neuroimaging.

[10] Huntley J., Bor D., Deng F., Mancuso M., Mediano P.A.M., Naci L., Owen A.M., Rocchi L., Sternin A., Howard R. (2023). Assessing Awareness in Severe Alzheimer’s Disease. Front. Hum. Neurosci..

[11] Owen A.M., Coleman M.R., Boly M., Davis M.H., Laureys S., Pickard J.D. (2006). Detecting Awareness in the Vegetative State. Science.

[12] Monti M.M., Vanhaudenhuyse A., Coleman M.R., Boly M., Pickard J.D., Tshibanda L., Owen A.M., Laureys S. (2010). Willful Modulation of Brain Activity in Disorders of Consciousness. N. Engl. J. Med..

[13] Cruse D., Chennu S., Chatelle C., Bekinschtein T.A., Fernández-Espejo D., Pickard J.D., Laureys S., Owen A.M. (2011). Bedside Detection of Awareness in the Vegetative State: A Cohort Study. Lancet.

[14] Goldfine A.M., Bardin J.C., Noirhomme Q., Fins J.J., Schiff N.D., Victor J.D. (2013). Reanalysis of “Bedside Detection of Awareness in the Vegetative State: A Cohort Study”. Lancet.

[15] Cruse D., Chennu S., Chatelle C., Bekinschtein T.A., Fernández-Espejo D., Pickard J.D., Laureys S., Owen A.M. (2013). Reanalysis of “Bedside Detection of Awareness in the Vegetative State: A Cohort Study”—Authors’ Reply. Lancet.

[16] Wolpaw J.R., Birbaumer N., Heetderks W.J., McFarland D.J., Peckham P.H., Schalk G., Donchin E., Quatrano L.A., Robinson C.J., Vaughan T.M. (2000). Brain-Computer Interface Technology: A Review of the First International Meeting. IEEE Trans. Rehabil. Eng..

[17] Sellers E.W., Donchin E. (2006). A P300-Based Brain–Computer Interface: Initial Tests by ALS Patients. Clin. Neurophysiol..

[18] Nijboer F., Sellers E.W., Mellinger J., Jordan M.A., Matuz T., Furdea A., Halder S., Mochty U., Krusienski D.J., Vaughan T.M. (2008). A P300-Based Brain–Computer Interface for People with Amyotrophic Lateral Sclerosis. Clin. Neurophysiol..

[19] Silvoni S., Volpato C., Cavinato M., Marchetti M., Priftis K., Merico A., Tonin P., Koutsikos K., Beverina F., Piccione F. (2009). P300-Based Brain-Computer Interface Communication: Evaluation and Follow-up in Amyotrophic Lateral Sclerosis. Front. Neurosci..

[20] Toppi J., Quattrociocchi I., Riccio A., D’Ippolito M., Aloisi M., Colamarino E., Pichiorri F., Cincotti F., Formisano R., Mattia D. (2024). EEG-Derived Markers to Improve Prognostic Evaluation of Disorders of Consciousness. IEEE J. Biomed. Health Inform..

[21] Yang H., Wu H., Kong L., Luo W., Xie Q., Pan J., Quan W., Hu L., Li D., Wu X. (2024). Precise Detection of Awareness in Disorders of Consciousness Using Deep Learning Framework. Neuroimage.

[22] He Y., Wang N., Liu D., Peng H., Yin S., Wang X., Wang Y., Yang Y., Si J. (2024). Assessment of Residual Awareness in Patients with Disorders of Consciousness Using Functional Near-Infrared Spectroscopy–Based Connectivity: A Pilot Study. Neurophotonics.

[23] Abdalmalak A., Milej D., Norton L., Debicki D.B., Owen A.M., Lawrence K.S. (2021). The Potential Role of FNIRS in Evaluating Levels of Consciousness. Front. Hum. Neurosci..

[24] Page M.J., McKenzie J.E., Bossuyt P.M., Boutron I., Hoffmann T.C., Mulrow C.D., Shamseer L., Tetzlaff J.M., Akl E.A., Brennan S.E. (2021). The PRISMA 2020 Statement: An Updated Guideline for Reporting Systematic Reviews. Syst. Rev..

[25] Whiting P.F., Rutjes A.W.S., Westwood M.E., Mallett S., Deeks J.J., Reitsma J.B., Leeflang M.M.G., Sterne J.A.C., Bossuyt P.M.M. (2011). QUADAS-2: A Revised Tool for the Quality Assessment of Diagnostic Accuracy Studies. Ann. Intern. Med..

[26] Casali A.G., Gosseries O., Rosanova M., Boly M., Sarasso S., Casali K.R., Casarotto S., Bruno M.-A., Laureys S., Tononi G. (2013). A Theoretically Based Index of Consciousness Independent of Sensory Processing and Behavior. Sci. Transl. Med..

[27] Casarotto S., Comanducci A., Rosanova M., Sarasso S., Fecchio M., Napolitani M., Pigorini A., Casali A.G., Trimarchi P.D., Boly M. (2016). Stratification of Unresponsive Patients by an Independently Validated Index of Brain Complexity. Ann. Neurol..

[28] Vanhaudenhuyse A., Noirhomme Q., Tshibanda L.J.-F., Bruno M.-A., Boveroux P., Schnakers C., Soddu A., Perlbarg V., Ledoux D., Brichant J.-F. (2010). Default Network Connectivity Reflects the Level of Consciousness in Non-Communicative Brain-Damaged Patients. Brain.

[29] Fernández-Espejo D., Soddu A., Cruse D., Palacios E.M., Junque C., Vanhaudenhuyse A., Rivas E., Newcombe V., Menon D.K., Pickard J.D. (2012). A Role for the Default Mode Network in the Bases of Disorders of Consciousness. Ann. Neurol..

[30] Demertzi A., Antonopoulos G., Heine L., Voss H.U., Crone J.S., de Los Angeles C., Bahri M.A., Di Perri C., Vanhaudenhuyse A., Charland-Verville V. (2015). Intrinsic Functional Connectivity Differentiates Minimally Conscious from Unresponsive Patients. Brain.

[31] Wu X., Zou Q., Hu J., Tang W., Mao Y., Gao L., Zhu J., Jin Y., Wu X., Lu L. (2015). Intrinsic Functional Connectivity Patterns Predict Consciousness Level and Recovery Outcome in Acquired Brain Injury. J. Neurosci..

[32] Cao B., Chen Y., Yu R., Chen L., Chen P., Weng Y., Chen Q., Song J., Xie Q., Huang R. (2019). Abnormal Dynamic Properties of Functional Connectivity in Disorders of Consciousness. Neuroimage Clin..

[33] Li M., Yang Y., Zhang Y., Gao Y., Jing R., Dang Y., Chen X., He J., Si J. (2021). Detecting Residual Awareness in Patients with Prolonged Disorders of Consciousness: An FNIRS Study. Front. Neurol..

[34] Si J., Yang Y., Xu L., Xu T., Liu H., Zhang Y., Jing R., Li J., Wang D., Wu S. (2023). Evaluation of Residual Cognition in Patients with Disorders of Consciousness Based on Functional Near-Infrared Spectroscopy. Neurophotonics.

[35] Liu Y., Kang X., Chen B., Song C., Liu Y., Hao J., Yuan F., Jiang W. (2023). Detecting Residual Brain Networks in Disorders of Consciousness: A Resting-State FNIRS Study. Brain Res..

[36] Kazazian K., Abdalmalak A., Novi S.L., Norton L., Moulavi-Ardakani R., Kolisnyk M., Gofton T.E., Mesquita R.C., Owen A.M., Debicki D.B. (2024). Functional Near-Infrared Spectroscopy: A Novel Tool for Detecting Consciousness after Acute Severe Brain Injury. Proc. Natl. Acad. Sci. USA.

[37] Wang Y., Zeng W., Zou L., Wang Q., Ren B., Xiong Q., Bai Y., Feng Z. (2025). Detecting Cognitive Motor Dissociation by Functional Near-Infrared Spectroscopy. Front. Neurol..

[38] Lulé D., Noirhomme Q., Kleih S.C., Chatelle C., Halder S., Demertzi A., Bruno M.-A., Gosseries O., Vanhaudenhuyse A., Schnakers C. (2013). Probing Command Following in Patients with Disorders of Consciousness Using a Brain–Computer Interface. Clin. Neurophysiol..

[39] Pan J., Xie Q., He Y., Wang F., Di H., Laureys S., Yu R., Li Y. (2014). Detecting Awareness in Patients with Disorders of Consciousness Using a Hybrid Brain–Computer Interface. J. Neural Eng..

[40] McCane L.M., Sellers E.W., McFarland D.J., Mak J.N., Carmack C.S., Zeitlin D., Wolpaw J.R., Vaughan T.M. (2014). Brain-Computer Interface (BCI) Evaluation in People with Amyotrophic Lateral Sclerosis. Amyotroph. Lateral Scler. Front. Degener..

[41] Wolpaw J.R., Bedlack R.S., Reda D.J., Ringer R.J., Banks P.G., Vaughan T.M., Heckman S.M., McCane L.M., Carmack C.S., Winden S. (2018). Independent Home Use of a Brain-Computer Interface by People with Amyotrophic Lateral Sclerosis. Neurology.

[42] Vansteensel M.J., Leinders S., Branco M.P., Crone N.E., Denison T., Freudenburg Z.V., Geukes S.H., Gosselaar P.H., Raemaekers M., Schippers A. (2024). Longevity of a Brain–Computer Interface for Amyotrophic Lateral Sclerosis. N. Engl. J. Med..

[43] Vansteensel M.J., Pels E.G.M., Bleichner M.G., Branco M.P., Denison T., Freudenburg Z.V., Gosselaar P., Leinders S., Ottens T.H., Van Den Boom M.A. (2016). Fully Implanted Brain–Computer Interface in a Locked-In Patient with ALS. N. Engl. J. Med..

[44] Lee M.-S., Lee S.-H., Moon E.-O., Moon Y.-J., Kim S., Kim S.-H., Jung I.-K. (2013). Neuropsychological Correlates of the P300 in Patients with Alzheimer’s Disease. Prog. Neuropsychopharmacol. Biol. Psychiatry.

[45] Parra M.A., Ascencio L.L., Urquina H.F., Manes F., Ibáñez A.M. (2012). P300 and Neuropsychological Assessment in Mild Cognitive Impairment and Alzheimer Dementia. Front. Neurol..

[46] Kim J.-S., Oh Y.-S., Lee K.-S., Kim Y.-I., Yang D.-W., Goldstein D.S. (2012). Association of Cognitive Dysfunction with Neurocirculatory Abnormalities in Early Parkinson Disease. Neurology.

[47] Longardner K., Bayram E., Litvan I. (2020). Orthostatic Hypotension Is Associated with Cognitive Decline in Parkinson Disease. Front. Neurol..

[48] Lee M., Laureys S. (2024). Artificial Intelligence and Machine Learning in Disorders of Consciousness. Curr. Opin. Neurol..

[49] Bonanno M., Cardile D., Liuzzi P., Celesti A., Micali G., Corallo F., Quartarone A., Tomaiuolo F., Calabrò R.S. (2025). Can Artificial Intelligence Improve the Diagnosis and Prognosis of Disorders of Consciousness? A Scoping Review. Front. Artif. Intell..

[50] Mou C., Zhao J., Yan Z., Zhang L., Tian X., Wang Y., Wang H., Hu J., He Z., Ling Y. (2026). An Explainable Multimodal Machine Learning Model for Diagnosing Disorders of Consciousness: Evidence from a Large Multicenter Chinese Cohort. J. Transl. Med..

[51] Manasova D., Belloli L.M.L., Rosenfelder M.J., Willacker L., Fló Rama E., Valota C., Hermann B., Kaufmann B.C., Pirastru A., Derchi C.C. (2026). Multimodal Multicentre Investigation of Diagnostic and Prognostic Markers in Disorders of Consciousness. Brain.

[52] Zonca L., Escrichs A., Patow G., Manasova D., Sanz-Perl Y., Annen J., Gosseries O., Laureys S., Sitt J.D., Deco G. (2025). Personalized Models of Disorders of Consciousness Reveal Complementary Roles of Connectivity and Local Parameters in Diagnosis and Prognosis. PLoS ONE.

[53] Wu H., Huang X., Lin D., Liao Z., Chen Z., Zhong H., Xu C., Jiang L., Xu N., Yang L. (2025). A New Multimodal Neuroprognostic Model for Chronic Disorders of Consciousness: Integrating Behavioral, Hormonal, and Imaging Features. Neuroimage.

[54] Amiri M., Raimondo F., Fisher P.M., Cacic Hribljan M., Sidaros A., Othman M.H., Zibrandtsen I., Bergdal O., Fabritius M.L., Hansen A.E. (2024). Multimodal Prediction of 3- and 12-Month Outcomes in ICU Patients with Acute Disorders of Consciousness. Neurocrit. Care.

[55] Kim J.S., Han J.W., Bae J.B., Moon D.G., Shin J., Kong J.E., Lee H., Yang H.W., Lim E., Kim J.Y. (2022). Deep Learning-Based Diagnosis of Alzheimer’s Disease Using Brain Magnetic Resonance Images: An Empirical Study. Sci. Rep..

[56] Kim M., Youn Y.C., Paik J. (2023). Deep Learning-Based EEG Analysis to Classify Normal, Mild Cognitive Impairment, and Dementia: Algorithms and Dataset. Neuroimage.

[57] Wang C., Tachimori H., Yamaguchi H., Sekiguchi A., Li Y., Yamashita Y. (2024). A Multimodal Deep Learning Approach for the Prediction of Cognitive Decline and Its Effectiveness in Clinical Trials for Alzheimer’s Disease. Transl. Psychiatry.

[58] Faugeras F., Rohaut B., Weiss N., Bekinschtein T., Galanaud D., Puybasset L., Bolgert F., Sergent C., Cohen L., Dehaene S. (2012). Event Related Potentials Elicited by Violations of Auditory Regularities in Patients with Impaired Consciousness. Neuropsychologia.

[59] King J.R., Faugeras F., Gramfort A., Schurger A., El Karoui I., Sitt J.D., Rohaut B., Wacongne C., Labyt E., Bekinschtein T. (2013). Single-Trial Decoding of Auditory Novelty Responses Facilitates the Detection of Residual Consciousness. Neuroimage.

[60] Sitt J.D., King J.-R., El Karoui I., Rohaut B., Faugeras F., Gramfort A., Cohen L., Sigman M., Dehaene S., Naccache L. (2014). Large Scale Screening of Neural Signatures of Consciousness in Patients in a Vegetative or Minimally Conscious State. Brain.

[61] Chennu S., Finoia P., Kamau E., Allanson J., Williams G.B., Monti M.M., Noreika V., Arnatkeviciute A., Canales-Johnson A., Olivares F. (2014). Spectral Signatures of Reorganised Brain Networks in Disorders of Consciousness. PLoS Comput. Biol..

[62] Vansteensel M.J., Klein E., van Thiel G., Gaytant M., Simmons Z., Wolpaw J.R., Vaughan T.M. (2023). Towards Clinical Application of Implantable Brain–Computer Interfaces for People with Late-Stage ALS: Medical and Ethical Considerations. J. Neurol..

[63] McCane L.M., Heckman S.M., McFarland D.J., Townsend G., Mak J.N., Sellers E.W., Zeitlin D., Tenteromano L.M., Wolpaw J.R., Vaughan T.M. (2015). P300-Based Brain-Computer Interface (BCI) Event-Related Potentials (ERPs): People with Amyotrophic Lateral Sclerosis (ALS) vs. Age-Matched Controls. Clin. Neurophysiol..

[64] Kim M.S., Yoon J.H., Hong J.M. (2018). Early Differentiation of Dementia with Lewy Bodies and Alzheimer’s Disease: Heart Rate Variability at Mild Cognitive Impairment Stage. Clin. Neurophysiol..

[65] Wang N., He Y., Zhu S., Liu D., Chai X., He Q., Cao T., He J., Li J., Si J. (2025). Functional Near-Infrared Spectroscopy for the Assessment and Treatment of Patients with Disorders of Consciousness. Front. Neurol..

[66] Fernández-Espejo D., Bekinschtein T., Monti M.M., Pickard J.D., Junque C., Coleman M.R., Owen A.M. (2011). Diffusion Weighted Imaging Distinguishes the Vegetative State from the Minimally Conscious State. Neuroimage.

[67] Stafford C.A., Owen A.M., Fernández-Espejo D. (2019). The Neural Basis of External Responsiveness in Prolonged Disorders of Consciousness. Neuroimage Clin..

[68] Crone J.S., Schurz M., Höller Y., Bergmann J., Monti M., Schmid E., Trinka E., Kronbichler M. (2015). Impaired Consciousness Is Linked to Changes in Effective Connectivity of the Posterior Cingulate Cortex within the Default Mode Network. Neuroimage.

[69] Riganello F., Larroque S.K., Bahri M.A., Heine L., Martial C., Carrière M., Charland-Verville V., Aubinet C., Vanhaudenhuyse A., Chatelle C. (2018). A Heartbeat Away from Consciousness: Heart Rate Variability Entropy Can Discriminate Disorders of Consciousness and Is Correlated with Resting-State FMRI Brain Connectivity of the Central Autonomic Network. Front. Neurol..

[70] Riganello F., Vatrano M., Cortese M.D., Tonin P., Soddu A. (2024). Central Autonomic Network and Early Prognosis in Patients with Disorders of Consciousness. Sci. Rep..

[71] Riganello F., Cortese M.D., Vatrano M., Lucca L.F., Soddu A. (2025). Autonomic Heart Rate Variability Trends Predict Outcome in Disorders of Consciousness. Sci. Rep..

[72] Liuzzi P., Campagnini S., Hakiki B., Burali R., Scarpino M., Macchi C., Cecchi F., Mannini A., Grippo A. (2023). Heart Rate Variability for the Evaluation of Patients with Disorders of Consciousness. Clin. Neurophysiol..

[73] Angerer M., Wilhelm F.H., Liedlgruber M., Pichler G., Angerer B., Scarpatetti M., Blume C., Schabus M. (2022). Does the Heart Fall Asleep?—Diurnal Variations in Heart Rate Variability in Patients with Disorders of Consciousness. Brain Sci..

[74] Liu K.Y., Elliott T., Knowles M., Howard R. (2022). Heart Rate Variability in Relation to Cognition and Behavior in Neurodegenerative Diseases: A Systematic Review and Meta-Analysis. Ageing Res. Rev..

[75] Ma Y., Zhang Y., Hamaya R., Westerhof B.E., Shaltout H.A., Kavousi M., Mattace-Raso F., Hofman A., Wolters F.J., Lipsitz L.A. (2025). Baroreflex Sensitivity and Long-Term Dementia Risk in Older Adults. Hypertension.

[76] Hermann B., Stender J., Habert M.-O., Kas A., Denis-Valente M., Raimondo F., Pérez P., Rohaut B., Sitt J.D., Naccache L. (2021). Multimodal FDG-PET and EEG Assessment Improves Diagnosis and Prognostication of Disorders of Consciousness. Neuroimage Clin..

[77] Yu Y., Meng F., Zhang L., Liu X., Wu Y., Chen S., Tan X., Li X., Kuang S., Sun Y. (2021). A Multi-Domain Prognostic Model of Disorder of Consciousness Using Resting-State FMRI and Laboratory Parameters. Brain Imaging Behav..

[78] Piccione F., Giorgi F., Tonin P., Priftis K., Giove S., Silvoni S., Palmas G., Beverina F. (2006). P300-Based Brain Computer Interface: Reliability and Performance in Healthy and Paralysed Participants. Clin. Neurophysiol..

[79] Arvaneh M., Robertson I.H., Ward T.E. (2019). A P300-Based Brain-Computer Interface for Improving Attention. Front. Hum. Neurosci..

[80] Liberati G., Birbaumer N. (2012). Using Brain–Computer Interfaces to Overcome the Extinction of Goal-Directed Thinking in Minimally Conscious State Patients. Cogn. Process..

[81] Li Y., Pan J., He Y., Wang F., Laureys S., Xie Q., Yu R. (2015). Detecting Number Processing and Mental Calculation in Patients with Disorders of Consciousness Using a Hybrid Brain-Computer Interface System. BMC Neurol..

[82] Curley W.H., Forgacs P.B., Voss H.U., Conte M.M., Schiff N.D. (2018). Characterization of EEG Signals Revealing Covert Cognition in the Injured Brain. Brain.

[83] Li J., Huang B., Wang F., Xie Q., Xu C., Huang H., Pan J. (2022). A Potential Prognosis Indicator Based on P300 Brain–Computer Interface for Patients with Disorder of Consciousness. Brain Sci..

[84] Chu C., Luo J., Tian X., Han X., Guo S. (2021). A P300 Brain-Computer Interface Paradigm Based on Electric and Vibration Simple Command Tactile Stimulation. Front. Hum. Neurosci..

[85] Maza A., Goizueta S., Dolores Navarro M., Noé E., Ferri J., Naranjo V., Llorens R. (2024). EEG-Based Responses of Patients with Disorders of Consciousness and Healthy Controls to Familiar and Non-Familiar Emotional Videos. Clin. Neurophysiol..

[86] Qu S., Wu X., Huang L., Zhou Y., Sun Q., Zeng F. (2026). Diagnosis of Disorders of Consciousness Using Nonlinear Feature Derived EEG Topographic Maps via Deep Learning. Sci. Rep..

[87] Della Bella G.A., Zang D., Gui P., Mateos D.M., Sitt J.D., Bekinschtein T.A., Manasova D., Sarton B., Ferre F., Silva S. (2025). Detection of EEG Dynamic Complex Patterns in Disorders of Consciousness. Commun. Biol..

[88] Altmayer V., Sangare A., Calligaris C., Puybasset L., Perlbarg V., Naccache L., Sitt J.D., Rohaut B. (2024). Functional and Structural Brain Connectivity in Disorders of Consciousness. Brain Struct. Funct..

[89] Liuzzi P., Magliacano A., De Bellis F., Mannini A., Estraneo A. (2022). Predicting Outcome of Patients with Prolonged Disorders of Consciousness Using Machine Learning Models Based on Medical Complexity. Sci. Rep..

[90] Willacker L., Raiser T.M., Bassi M., Bender A., Comanducci A., Rosanova M., Sobel N., Arzi A., Belloli L., Casarotto S. (2022). PerBrain: A Multimodal Approach to Personalized Tracking of Evolving State-of-Consciousness in Brain-Injured Patients: Protocol of an International, Multicentric, Observational Study. BMC Neurol..

[91] Yan S., Li Q., Li R., Zhang L., Zhang R., Chen M., Li M., Li R., Zhang H., Shi L. (2026). Prognosis Prediction of Patients with Disorders of Consciousness Based on Digital Twin Brain Models. J. Neuroeng. Rehabil..

[92] Ma Y., Bland J.K.S., Fujinami T. (2024). Classification of Alzheimer’s Disease and Frontotemporal Dementia Using Electroencephalography to Quantify Communication between Electrode Pairs. Diagnostics.

[93] Pfurtscheller G. (2010). The Hybrid BCI. Front. Neurosci..

[94] Riganello F., Candelieri A., Quintieri M., Conforti D., Dolce G. (2010). Heart Rate Variability: An Index of Brain Processing in Vegetative State? An Artificial Intelligence, Data Mining Study. Clin. Neurophysiol..

[95] Zhang X.-Y., Li J.-J., Lu H.-T., Teng W.-J., Liu S.-H. (2021). Positive Effects of Music Therapist’s Selected Auditory Stimulation on the Autonomic Nervous System of Patients with Disorder of Consciousness: A Randomized Controlled Trial. Neural Regen. Res..

[96] Goizueta S., Maza A., Sierra A., Navarro M.D., Noé E., Ferri J., Llorens R. (2025). Heart Rate Variability Responses to Personalized and Non-Personalized Affective Videos. A Study on Healthy Subjects and Patients with Disorders of Consciousness. Front. Psychol..

[97] Nicolini P., Lucchi T., Abbate C., Inglese S., Tomasini E., Mari D., Rossi P.D., Vicenzi M. (2022). Autonomic Function Predicts Cognitive Decline in Mild Cognitive Impairment: Evidence from Power Spectral Analysis of Heart Rate Variability in a Longitudinal Study. Front. Aging Neurosci..

[98] Meel-van den Abeelen A.S.S., Lagro J., Gommer E.D., Reulen J.P.H., Claassen J.A.H.R. (2013). Baroreflex Function Is Reduced in Alzheimer’s Disease: A Candidate Biomarker?. Neurobiol. Aging.

[99] Saibene M., Gu Y., Ballegaard M., Andersen T.S., Bardram J.E., Puthusserypady S. (2026). Translational Perspectives on Brain-Heart Interplay: From Methodologies to Clinical Applications. Comput. Biol. Med..

[100] Nicolini P., Mari D., Abbate C., Inglese S., Bertagnoli L., Tomasini E., Rossi P.D., Lombardi F. (2020). Autonomic Function in Amnestic and Non-Amnestic Mild Cognitive Impairment: Spectral Heart Rate Variability Analysis Provides Evidence for a Brain–Heart Axis. Sci. Rep..

[101] Rosazza C., Andronache A., Sattin D., Bruzzone M.G., Marotta G., Nigri A., Ferraro S., Rossi Sebastiano D., Porcu L., Bersano A. (2016). Multimodal Study of Default-mode Network Integrity in Disorders of Consciousness. Ann. Neurol..

[102] Song M., Yang Y., He J., Yang Z., Yu S., Xie Q., Xia X., Dang Y., Zhang Q., Wu X. (2018). Prognostication of Chronic Disorders of Consciousness Using Brain Functional Networks and Clinical Characteristics. eLife.

[103] Zhang J., Zhang E., Yuan C., Zhang H., Wang X., Yan F., Pei Y., Li Y., Wei M., Yang Z. (2022). Abnormal Default Mode Network Could Be a Potential Prognostic Marker in Patients with Disorders of Consciousness. Clin. Neurol. Neurosurg..

[104] Kazazian K., Monti M.M., Owen A.M. (2025). Functional Neuroimaging in Disorders of Consciousness: Towards Clinical Implementation. Brain.

[105] Zhao S., Cao Y., Yang W., Yu J., Xu C., Dai W., Li S., Pan G., Luo B. (2024). DOCTer: A Novel EEG-Based Diagnosis Framework for Disorders of Consciousness. J. Neural Eng..

[106] Oxley T.J., Yoo P.E., Rind G.S., Ronayne S.M., Lee C.M.S., Bird C., Hampshire V., Sharma R.P., Morokoff A., Williams D.L. (2021). Motor Neuroprosthesis Implanted with Neurointerventional Surgery Improves Capacity for Activities of Daily Living Tasks in Severe Paralysis: First in-Human Experience. J. Neurointerv. Surg..

[107] Loser V., Rossetti A.O., Rasic M., Novy J., Schindler K.A., Rüegg S., Alvarez V., Beuchat I. (2024). Relevance of Continuous EEG versus Routine EEG for Outcome Prediction after Traumatic Brain Injury. Eur. Neurol..

[108] Wang L., Wu Q., Yang Z., Yang Y., Luo Y., Cao Y., Wu L., Xie Y., Wang Y. (2022). Preliminary Study of Vagus Nerve Magnetic Modulation in Patients with Prolonged Disorders of Consciousness. Neuropsychiatr. Dis. Treat..

[109] Dong X., Tang Y., Zhou Y., Feng Z. (2023). Stimulation of Vagus Nerve for Patients with Disorders of Consciousness: A Systematic Review. Front. Neurosci..

[110] Zhou Y.-F., Kang J.-W., Xiong Q., Feng Z., Dong X.-Y. (2023). Transauricular Vagus Nerve Stimulation for Patients with Disorders of Consciousness: A Randomized Controlled Clinical Trial. Front. Neurol..

[111] Zhang J.J., Lo Y.T., Wee A.X.Q., Yeo J., Suan E., Zhang Z., Lim M.J.R., Chua K.S.G. (2026). An Individual Patient Data Meta-Analysis on Vagal Nerve Stimulation for Recovery from Disorders of Consciousness. Sci. Rep..

[112] Osińska A., Rynkiewicz A., Binder M., Komendziński T., Borowicz A., Leszczyński A. (2022). Non-Invasive Vagus Nerve Stimulation in Treatment of Disorders of Consciousness—Longitudinal Case Study. Front. Neurosci..

[113] Li Y., Riganello F., Yu J., Vatrano M., Shen M., Cheng L., Hu X., Ni C., Wang F., Zheng B. (2025). The Autonomic Response Following TaVNS Predicts Changes in Level of Consciousness in DoC Patients. Sci. Rep..

